# Multiomics analysis of polyamine metabolism in colorectal cancer, highlighting the key role of extracellular putrescine in impairing CXCR6^+^CD8^+^ T cell anti-tumor activity

**DOI:** 10.7717/peerj.20663

**Published:** 2026-02-12

**Authors:** Ziru Tan, Yuqiang Zhang, Cong pei jia, Haifeng Lian, Bin Wang

**Affiliations:** 1 Department of Gastroenterology and Institute of Digestive Disease, Binzhou Medical University Hospital, Binzhou, China; 2 Yantai Affiliated Hospital of Binzhou Medical University, Department of Clinical Laboratory, Yantai, China; 3 Department of Laboratory Medicine, The First Affiliated Hospital of Xi’an Jiaotong University, Xi’an, China; 4 Department of Gastroenterology, Yantai Affiliated Hospital of Binzhou Medical University, Yantai, China; 5 Department of Immunology, Binzhou Medical University, Binzhou, China

**Keywords:** CXCR6+CD8+ T cells, Putrescine metabolisms, Colorectal cancer, Cytotoxic activity, ODC1

## Abstract

The contribution of putrescine (PUT) metabolism to impaired tumor immunosurveillance in colorectal cancer (CRC) requires a thorough examination of its biosynthesis, catabolism, and transport during tumor development. This study found, unexpectedly, that a better prognosis was associated with higher expression of the PUT biosynthesis genes *ODC1* and *AGMAT*, while elevated expression of biosynthesis inhibitors and transport genes predicted worse outcomes. Two independent cohorts were deconvoluted by integrating data from 517,191 cells across 221 samples from 131 subjects. Higher percentages of epithelial subpopulations with low PUT transport scores were linked to improved prognosis. In contrast, greater proportions of T/natural killer/ILC cells with low biosynthesis but relatively higher transport scores were associated with poorer outcomes. PUT supplementation in HCT-116 and RKO cells promoted the tumor cell proliferation, but had no effect on cell migration or the expression of N-cadherin and E-cadherin. CXCR6^+^ CD8^+^ T cells, which were more prevalent in tumor tissue, exhibited significantly higher cytotoxicity compared to CXCR6^−^ CD8^+^ T cells, as assessed using updated gene sets for T cell functional evaluation . However, CXCR6^+^CD8^+^ T cells also displayed elevated markers of exhaustion. Notably, higher CXCR6 expression and increased infiltration of CXCR6^+^ CD8^+^ T cells correlated with improved prognosis in both mismatch repair-proficient and mismatch repair-deficient CRC. To estimate the local accumulation of PUT around CXCR6^+^ CD8^+^ T cells, a novel “Pi value” was defined. A negative correlation was found between Pi values and both the cytotoxic activity and pro-inflammatory potential of these cells. Further investigation using a CRC tumor antigen-based *in vitro* system for the efficient induction of CXCR6^+^ CD8^+^ T cells revealed that extracellular PUT inhibits their cytotoxic function. Additionally, in a Dextran Sulfate Sodium (DSS)-induced colitis model combined with single-cell RNA sequencing, PUT supplementation resulted in the elimination of CXCR6^+^ CD8^+^ T cells in the colon. These findings provide new insights into how polyamine metabolism, particularly involving extracellular PUT, impairs the anti-tumor activity of CXCR6^+^CD8^+^ T cells, potentially contribut ing to CRC progression.

## Introduction

Colorectal cancer (CRC) is the third most common malignancy worldwide with an aggressive phenotype and poor prognosis (Bray et al., 2018; Piawah & Venook, 2019). Polyamine metabolism, including the metabolites putrescine (PUT), spermidine, and spermine, plays a critical role in tumor development, including CRC (Holbert, Casero & Stewart, 2024; Holbert et al., 2022). These metabolites promote tumor survival and shape a tumor-permissive microenvironment. Despite the interrelated biosynthetic pathways for PUT, spermidine, and spermine, their functional roles in intestinal diseases can be distinct and sometimes even contradictory. For example, studies on colitis have reported contradictory effects for PUT and spermidine (Gobert et al., 2022; Grosheva et al., 2020), with evidence supporting both exacerbating and protective effects. This inconsistency extends to Dextran Sulfate Sodium (DSS)-induced colitis (Grosheva et al., 2020; Nakamura et al., 2021), where PUT has been shown to either worsen or ameliorate the disease. Difluoromethylornithine (DFMO), an irreversible inhibitor of *ODC1*, the rate-limiting enzyme in PUT biosynthesis, has long been explored as a therapeutic agent in cancer. However, its clinical efficacy as monotherapy is limited, possibly due to compensatory increases in extracellular polyamine uptake by tumor cells (Holbert, Casero & Stewart, 2024). Therefore, a deeper understanding of polyamine metabolism—biosynthesis, catabolism, and transport—is essential to elucidate its role in CRC progression.

Impaired tumor surveillance is a key driver in CRC development, with CD8^+^ cytotoxic T cells as primary effectors. Chemokines and their receptors are essential for T cell activation, differentiation, and function (Laufer & Legler, 2018; Rahimi & Luster, 2018). Recent studies have emphasized the importance of C-X-C motif chemokine receptor 6 (CXCR6) in supporting the anti-tumor functions of cytotoxic CD8^+^ T cells. CXCR6 facilitates positioning of these cells within the tumor microenvironment (TME) (Di Pilato et al., 2021), thereby enhancing their survival and function. Compared to CXCR6^−^CD8^+^ T cells, CXCR6^+^CD8^+^ T cells exhibit enhanced immune activity and responsiveness to immune checkpoint blockade, which correlates with improved clinical outcomes (Muthuswamy et al., 2021; Wang et al., 2021). T-cell surveillance is influenced by a variety of factors, including chemokine and cytokine signaling, cytotoxicity, inflammation promotion, expression of co-inhibitory and co-stimulatory molecules, tissue residency, and exhaustion status. However, a comprehensive characterization of CXCR6^+^CD8^+^ T cell function in CRC, particularly using large cohorts, has been limited.

Polyamines play complex roles in T cell development and function, often influenced by the TME. While polyamines are essential for T cell development and activation—requiring increased uptake of polyamines and arginine (Wu et al., 2020)—deficiency in ODC disrupts CD4^+^ T cell lineage commitment (Puleston et al., 2021). Conversely, polyamines such as PUT can impair immune function. For example, PUT inhibits T follicular helper cell homing by downregulating CXCR4 (Chisolm et al., 2019), and spermidine can suppress TCR signaling (Hibino et al., 2023). Polyamines also influence myeloid cell polarization, with PUT suppressing M1 macrophage activation and intestinal inflammation through histone modifications (Hardbower et al., 2017). Previous work has shown that extracellular PUT enhances the regulatory phenotype of dendritic cells and suppresses germinal center B cell differentiation in Peyer’s patches (Huang et al., 2023; Wei et al., 2024). Additionally, PUT has been observed to promote epithelial-mesenchymal transition (EMT) in gastric cancer cells (Bi et al., 2024). Despite these insights, the specific effects of extracellular PUT on the tumor-surveillance function of CXCR6^+^CD8^+^ T cells in CRC remain poorly understood.

This study investigates the relationships between genes involved in PUT metabolism and CRC outcomes using integrative single-cell and bulk RNA sequencing (RNA-seq) data. The effects of extracellular PUT on CRC cell lines (HCT-116 and RKO) were examined, along with the surveillance capacity of CXCR6^+^CD8^+^ T cells. The functional association with polyamine metabolism was explored through multi-omics approaches and both *in vitro* and *in vivo* experiments. The findings provide valuable insights into the interplay between host and microbial polyamine metabolism and its influence on CRC progression.

## Materials and Methods

### Ethics statement

The animal experimental protocol was designed in strict adherence to the guidelines and was reviewed and approved by the Research Ethics Committee of Binzhou Medical University (BZMU2021-163). The study complied with the guidelines and regulations established by the same committee (BZMU2022-631). All donors provided written informed consent prior to participation. Consent forms and the original Institutional Review Board (IRB) approval documents are retained by the authors’ institution and are available upon reasonable request.

### Integration of single-cell RNA-seq (scRNA-seq) datasets

All datasets were uniformly normalized prior to integration. Single-cell RNA-seq (sc-RNA-seq) data were processed with SCTransform and integrated using the Harmony algorithm to remove platform-related variance. Bulk RNA-seq datasets were normalized to transcripts per million (TPM), log_2_-transformed, and *z*-score scaled, followed by batch correction using the ComBat method. Post-correction principal component analysis (PCA) and uniform manifold approximation and projection (UMAP) confirmed that clustering was driven by biological cell type rather than dataset origin. In this study, we downloaded seven scRNA-seq datasets (GSE132465, GSE144735, GSE161277, GSE166555, GSE188711, GSE200997, and GSE221575) from the Gene Expression Omnibus (GEO) database to broadly characterize CXCR6^+^CD8^+^ T cells. An additional scRNA-seq dataset (GSE178341) was downloaded to explore the potential role of CXCR6^+^CD8^+^ T cells in mismatch repair-proficient (MMRp) CRC. The Seurat package was used to read the expression matrices and perform downstream analyses. For each dataset, metadata were enriched with information such as dataset ID, patient ID, and tissue type (Tumor, Tumor Border, and Normal Tissue). The seven datasets were merged (integrated scRNA-seq) and then subjected to quality control. Low-quality cells were excluded based on the following criteria: mitochondrial gene ratio >15%, ribosomal gene read ratio >50%, hemoglobin gene ratio >1%, RNA read count (nCount_RNA) >10,000, and total feature count (nFeature_RNA) <400 or >6,000. For dataset GSE178341, the mitochondrial gene ratio threshold was set at 30%, and the nFeature_RNA maximum was 7,000, consistent with the original literature (Pelka et al., 2021). To minimize noise, mitochondrial genes (prefix “MT-”) and ribosomal genes (prefix “RPL” or “RPS”) were removed from the expression data (Xue et al., 2022). RPCA-based integration was used to combine the seven scRNA-seq datasets according to the recommended protocol. Dimensionality reduction was performed using UMAP, followed by neighborhood detection and clustering at resolutions ranging from 0.3 to 0.8. The AUC values of marker genes were calculated using the FindAllMarkers function with the test parameter.use = “roc” to identify the optimal resolution (Chaffin et al., 2022). Clusters were annotated to known cell types based on marker gene expression. Marker genes for CXCR6^+^CD8^+^ T cells were identified using ROC-based algorithms and differential expression percentages. Only genes with an area under the curve (AUC) >0.85 and an expression percentage difference >0.55 (CXCR6^+^CD8^+^ T cells *vs.* all other cells) were selected. Additionally, subpopulations within broad cell types were automatically identified using non-negative matrix factorization (NMF) and UMAP (resolution = 0.3). CXCR6^+^CD8^+^ T cells were defined based on the expression percentage of CXCR6 in CD8^+^ T cells. To correct for batch effects between scRNA-seq datasets, the Harmony integration algorithm implemented in Seurat v4 was used following standard normalization procedures. For bulk transcriptomic data, expression matrices were log_2_-transformed and z-score normalized to ensure comparability between The Cancer Genome Atlas (TCGA) and GEO cohorts.

### Bulk RNA-seq cohorts, deconvolution, and survival analysis

For bulk RNA-seq datasets, raw read counts were normalized to TPM, then log_2_-transformed and *z*-scored. Batch effects among datasets were evaluated using PCA and corrected with the ComBat function from the sva R package. The Cancer Genome Atlas Colon Adenocarcinoma Collection (TCGA-COAD) cohort was downloaded from the TCGA database. The expression matrix and survival data for cohort GSE39582 (*n* = 585) were obtained from the Gene Expression Omnibus database. The statistical power of this design, calculated using RNASeqPower, was 1. To deconvolute the two bulk RNA-seq datasets using the corresponding scRNA-seq references (integrated scRNA-seq for TCGA-COAD and GSE178341 for GSE39582), the high-dimensional scRNA-seq data were split into broad cell-type groups. For each type, cells were downsampled to 10,000 using sketching methods. Clustering was performed using NMF-based UMAP with rank = 10 and resolution = 1. Subpopulations were annotated based on broad cell types (*e.g.*, T cells, B cells, and Myeloid cells) and assigned cluster labels (*e.g.*, T_1, B_4). These annotated objects were merged to construct a scRNA-seq dataset suitable for use with the MuSiC2 package, which was used to estimate subpopulation proportions in the bulk RNA-seq datasets. Survival analysis was performed using Kaplan–Meier plots generated with the survival package.

### Gene set variation analysis, gene set enrichment analysis, and single-sample gene set enrichment analysis

**Figure 1:**
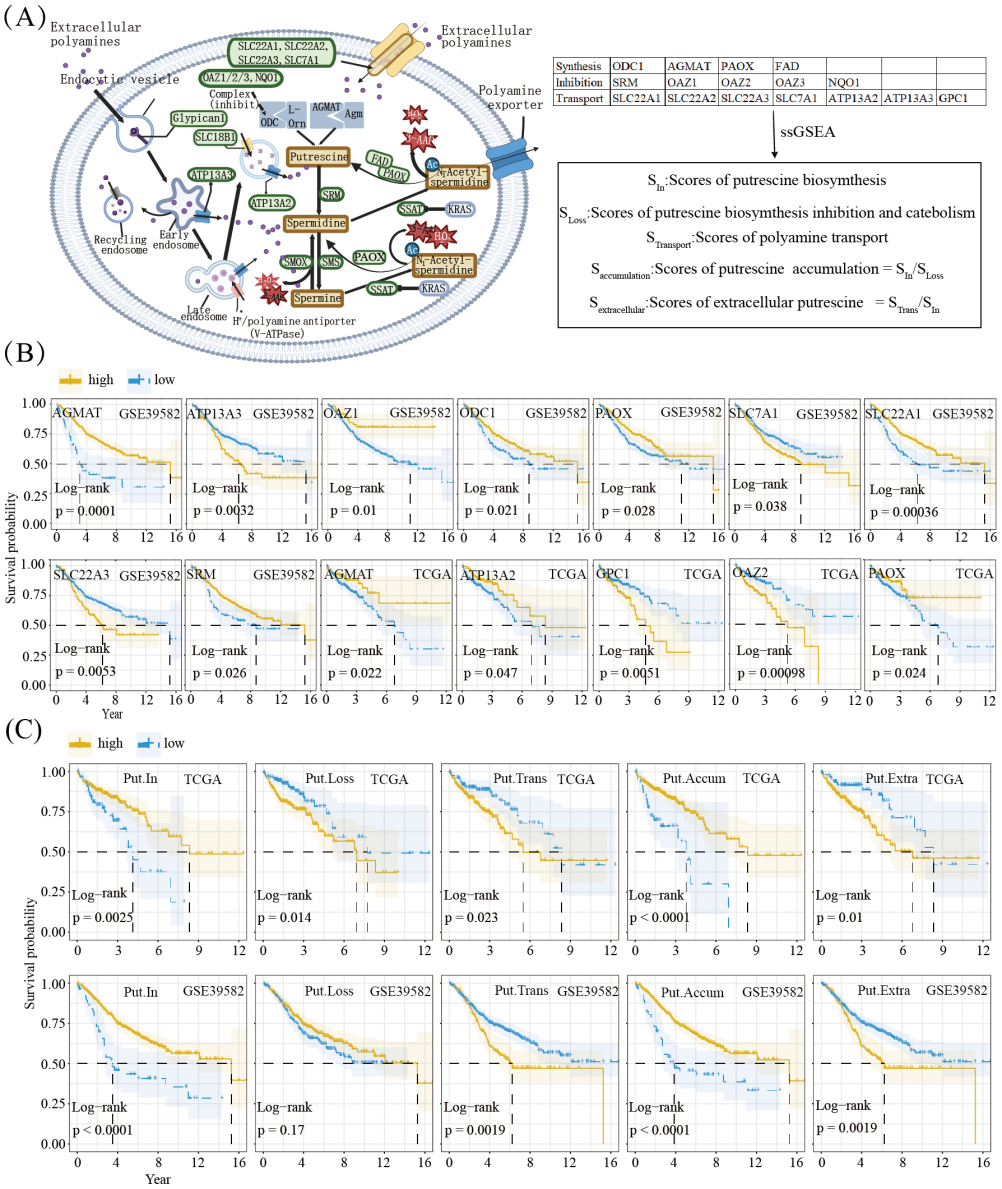
Associations of polyamine metabolism with the prognosis of CRC. (A) The left image shows the molecular mechanism of putrescine biosynthesis, metabolism, and transport pathways. The right image shows the flow chart of the scores calculated by the ssGSEA method for SIn, SLoss, and STrans, as well as the scores for Saccumulation and Sextracellular; Based on the TCGA-COAD and GSE39582 cohorts, Kaplan–Meier survival curves illustrate the associations between the prognosis and the putrescine metabolisms genes (Created in BioRender. tan, z. (2025) https://BioRender.com/gi37bq1) (B) or the GSVA scores of putrescine metabolisms gene sets (C). The genes or the gene sets were labeled in the front of the cohorts (*e.g.*, AGMAT GSE39582) at the top of the Kaplan-Meier curves. The log-rank p values were labeled in the left bottom of the curves.

For pathway-level analyses, gene set variation analysis (GSVA) was applied to normalized expression matrices, ensuring uniform data distribution across cohorts before downstream survival modeling. As illustrated in Fig. 1A, we categorized genes involved in PUT biosynthesis, catabolism, and transport into three gene sets based on their contribution to PUT content: Biosynthesis (ODC1, AGMAT, and PAOX), Loss (SRM, OAZ1/2/3, and NQO1), and Transport (SLC22A1, SLC22A2, SLC22A3, SLC7A1, ATP13A2, ATP13A3, and GPC1) (Table S1). single-sample gene set enrichment analysis (ssGSEA) from the GSVA package was applied to estimate PUT metabolism levels in the TCGA-COAD and GSE39582 cohorts. GSVA was also applied to the two scRNA-seq datasets to assess PUT metabolism in each cell. Differentially expressed genes (DEGs) were identified using the FindMarkers function in Seurat with min.pct = 0.3. Functional enrichment was performed using gene set enrichment analysis (GSEA) and GSVA against T cell-related gene sets tailored to the study objectives (Table S2), an updated version in our previous report (Shi et al., 2024).

### Associations between PUT metabolism and Kyoto Encyclopedia of Genes and Genomics (KEGG) pathways in epithelial cells *via* canonical correlation analysis

The GSVA scores for PUT metabolism and Kyoto Encyclopedia of Genes and Genomics (KEGG) pathways in epithelial cells were calculated using the GSVA package. Canonical correlation analysis (CCA) was implemented in the PAM package to assess the explanatory power of PUT metabolism for KEGG pathway activity. The top 20 KEGG pathways with the highest weights were extracted, and their Pearson correlations with PUT metabolism scores were computed.

### Estimating the association between PUT metabolism and CXCR6^+^**CD8**^+^ T cells

The average expression levels of ODC1, AGMAT, and PAOX were summed to define the expression score for PUT biosynthesis (Exp_in_). The score for PUT loss (Exp_loss_) was computed as the sum of average expressions of SRM, OAZ1/2/3, and NQO1. The PUT accumulation score (S_PA_) was calculated as Exp_in_/Exp_loss_. To estimate the surrounding PUT accumulation (Pi) for CXCR6^+^CD8^+^ T cells, the sum of S_PA_ scores in all non-CXCR6^+^CD8^+^ cells (S_1_) was divided by the number of CXCR6^+^CD8^+^ T cells (N): Pi = S_1_/N. Subsequently, associations between Pi and the median GSVA scores of T cell functional gene sets in CXCR6^+^CD8^+^ T cells were evaluated using a LASSO regression model across all samples.

### DSS-induced ulcerative colitis models and hematoxylin and eosin staining

The 6-week-old C57 mice were purchased from the Jinan Pengyue Experimental Animal Breeding Institute. The animal experiments were approved by the Institutional Animal Care and Use Committee of Binzhou Medical University and conducted in accordance with the 3R principles. The criteria for euthanizing the animals to minimize unnecessary suffering included severe pain, infection, unresponsiveness to treatment, and irreparable limb injuries impairing normal activities. None of the mice met these criteria during the study period.

A total of nine male C57BL/6 mice were randomly and equally divided into three groups: the control group receiving normal food and clean water (Control group), the putrescine group receiving 1% putrescine-supplemented food and 2.5% DSS (P/DSS group), and the DSS group receiving normal food and 2.5% DSS. Group assignment was performed using a random number table to ensure unbiased distribution. The sample size was based on commonly used sample sizes in the previous literature. All mice were kept under specific pathogen-free conditions for 10 days before the experiment. They were housed in individually ventilated cages under strict disinfection protocols, with controlled temperature (22 °C ± 2 °C), relative humidity (40%–70%), and a 12-hour light/dark cycle. Daily care and monitoring were consistent across groups, and feed and drinking water treatments were administered according to group allocation. On the eleventh day, mice that remained free of the specific pathogen were selected for further study. No accidental deaths occurred, and no mice were excluded. At the end of the experiment, mice were euthanized *via* cervical dislocation following isoflurane anesthesia. The absence of pupil reflex, heartbeat, and respiration confirmed death. Colons were harvested, rinsed three times in 4 °C PBS (0.1 M, pH = 7.2), and prepared into Swiss rolls. These tissue samples were fixed in 4% formalin, embedded in paraffin, sectioned, and stained with hematoxylin and eosin for histological analysis.

### scRNA-seq and data processing

The models were randomly reconstructed to obtain three colons per group. Lamina propria mononuclear cells (LPMCs) were isolated as previously described (Wei et al., 2024). Whole colonic cells were dissociated using the RWD Intestine Enzymolysis Kit (DHIE-5007), and equal numbers of LPMCs and entire cells (1:1) were combined to form a single-cell suspension. scRNA-seq libraries were prepared using the SeekOne^®^ Digital Droplet Single-Cell 3′Transcriptome Kit (SeekOne, Beijing, China) according to the manufacturer’s protocol and sequenced at Novogene Co., Ltd. (Beijing, China). Raw sequencing data were processed following previously published methods (Shi et al., 2024). Downstream analyses—including quality control, cluster annotation, and identification of DEGs—were conducted in R using the same methodology described earlier.

### *In vitro* culturing of HCT-116 and RKO cells and estimations of malignant behavior

HCT-116 cells were cultured in Dulbecco’s Modified Eagle Medium and RKO cells in MEM, supplemented with 10% fetal bovine serum (FBS) and 1% penicillin-streptomycin. In the PUT treatment group, PUT was added to the medium at a final concentration of five µg/mL. The malignant behavior of the cells was evaluated as described in a previous study (Bi et al., 2024). Following the manufacturer’s instructions, cell proliferation was measured using an MTT assay kit (Elabscience Biotechnology Co., Ltd.)(Elabscience Biotechnology Co., Ltd). Cell migration and invasion were assessed using a 24-well Boyden chamber with 8-µm pore-size polycarbonate membranes (Corning, Union City, CA, USA). Wound-healing assays were conducted by culturing cells at 37 °C under 5% carbon dioxide (CO_2_), followed by wound imaging at 0, 24, and 48 h. E-cadherin and N-cadherin expression levels in HCT-116 and RKO cells were determined *via* Western blot analysis. Although this study used HCT-116 and RKO cell lines, a previous related study used AGS and MKN-28 cells. Western blot procedures followed the previously published protocol, including reagents, antibodies (anti-Tubulin, anti-E-cadherin, and anti-N-cadherin), and dilution ratios (Bi et al., 2024).

### *In vitro* induction of CXCR6^+^CD8^+^T cells and cytotoxicity estimation

HCT-116 cells (10^7^ cells/mL) were subjected to five cycles of freezing in liquid nitrogen and thawing at 37 °C. The resulting lysate was centrifuged at 6,000 g for 30 min, and the supernatant was filtered using a 0.22µm sterile membrane (Nalgene, 720-1320). Peripheral blood mononuclear cells (PBMCs) were isolated from peripheral blood collected from healthy adult volunteers, after written informed consent was obtained. The study protocol was reviewed and approved by the Institutional Ethics Committee (Approval No. 2023-HSYY555). PBMCs were seeded in 24-well plates at 2 × 10^6^ cells/mL. These cells were stimulated with 30 μL/mL ImmunoCult™ Human CD3/CD28/CD2 T Cell Activator (Stemcell, Cat. 10990), 20 ng/mL recombinant human interleukin-15 (BioLegend, Cat. 570306), and 200 μL of BALL-1 cell antigen. The culture medium was RPMI 1640 supplemented with 10% FBS and 1% penicillin/streptomycin, and the culture was maintained at 37 °C in 5% CO_2_. During the induction period, IL-15 was added every other day, and the T Cell Activator was added weekly. Cells were passaged every 2–3 days to maintain optimal density. A final concentration of two µg/mL was added daily to the PUT group. Cell viability was assessed using Zombie NIR™ Fixable Viability Dye (Cat. 423106; BioLegend), and only live (Zombie NIR^−^) cells were included in subsequent analyses. Following stimulation, induction efficiency and cell phenotypes were assessed *via* flow cytometry at weeks 1 and 2.

### Statistical analysis

The Wilcoxon rank-sum test built into the analysis software was used for bioinformatics analyses. For all other comparisons, a one-way analysis of variance was performed using GraphPad Prism 8.4.3. *P* < 0.05 was considered statistically significant.

## Results

### Genes involved in PUT biosynthesis predict a good, favorable prognosis based on integrative omics

As shown in Fig. 1B, Kaplan–Meier (KM) plots for the TCGA-COAD and GSE39582 cohorts surprisingly indicate that genes involved in PUT synthesis (*ODC1*, *AGMAT*, *PAOX*) are associated with better prognosis. In contrast, genes involved in PUT catabolism and transport (*SRM*, *OAZ2*, *SLC22A3*, *SLC7A1*, *ATP13A3*, and *GPC1*) are associated with a poorer prognosis. GSVA scores for gene sets related to synthesis, inhibition, and transport were calculated using ssGSEA for both the TCGA-COAD and GSE39582 datasets (Fig. 1C). Higher GSVA scores for PUT synthesis genes were associated with improved prognostic outcomes. In contrast, inhibition of synthesis or increased transport correlated with poorer prognosis. Furthermore, intracellular PUT accumulation was estimated using the S_In_ and S_Loss_ metrics, with higher values indicating a better prognosis. In contrast, extracellular PUT levels, estimated using the S_Trans_/S_In_ ratio, were positively associated with a worse prognosis.

**Figure 2:**
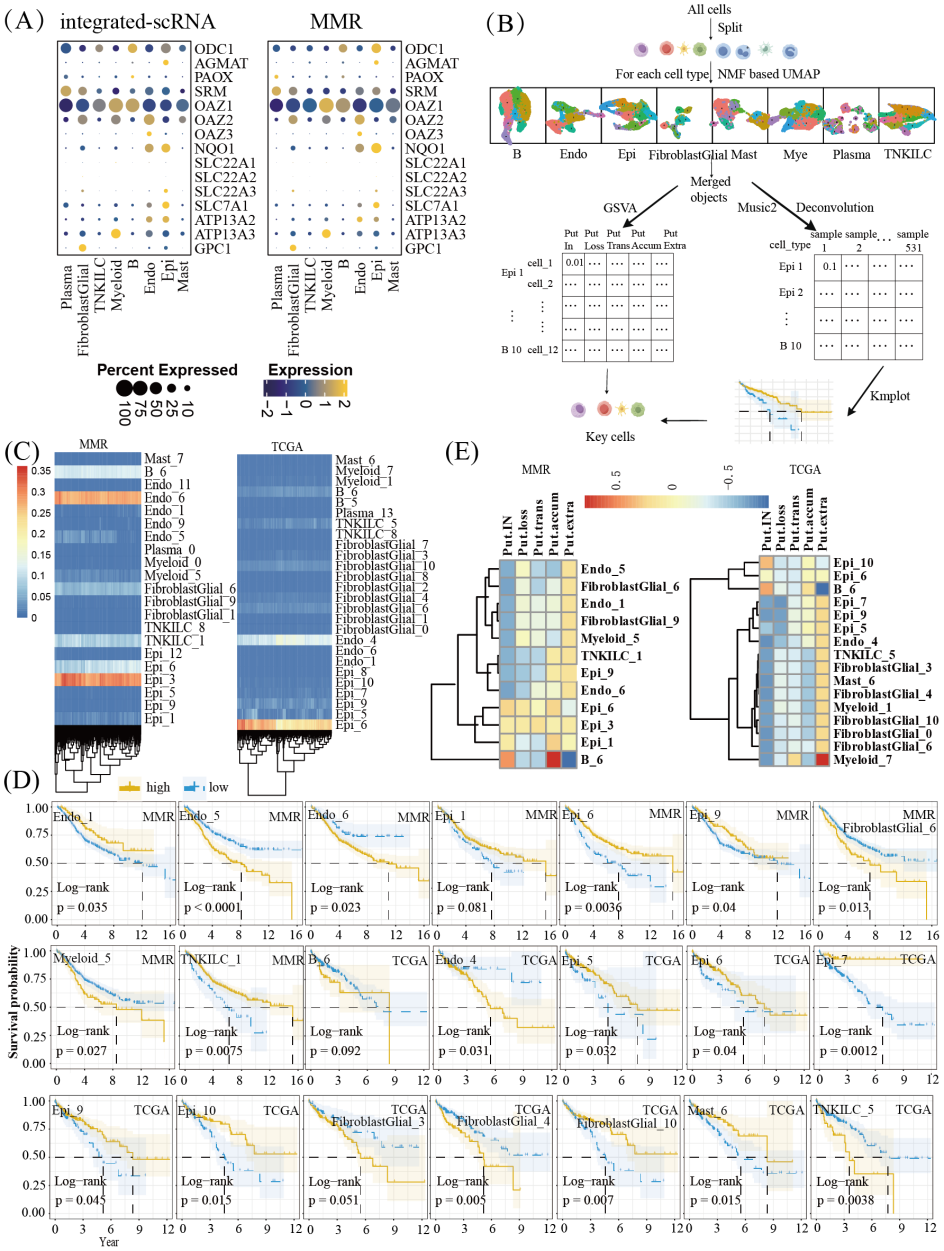
Analysis of putrescine metabolism-related gene expression and subpopulation identification in scRNA-seq data. (A) The expression status of putrescine metabolism-related genes in each subgroup in integrated-scRNA and MMR; (B) design of gene set exploration algorithms. The automatic identification of subpopulations for each major cell type using the NMF based UMAP method and the deconvoluting of subpopulation proportions by the MuSiC2 algorithm to estimate the prognostic value of subpopulations are demonstrated; (C) plot of deconvolution results between MMR and TCGA datasets; (D) survival curves of different cell subsets in the MMR and TCGA datasets; (E) heat map of the relationship between putrescine concentration and cell subtype.

We then examined PUT metabolism at the single-cell level using RNA-seq. The integrated scRNA-seq dataset, which incorporates seven public datasets (GSE132465, GSE144735, GSE161277, GSE166555, GSE188711, GSE200997, and GSE221575), comprised 231,640 cells from 121 samples across 69 subjects after quality filtering. Based on the expression of classical marker genes (Fig. S1), cells were broadly categorized into epithelial cells, endothelial cells, T, natural killer, and innate lymphoid cells (T/natural killer (NK)/innate lymphoid cells (ILC)), B cells, plasma cells, myeloid cells, mast cells, and fibroblasts mixed with glial cells. Similarly, 285,551 cells from 100 samples across 62 subjects in the GSE178341 (MMR) dataset were assigned to the same eight cell types (Fig. S2). As shown in Fig. 2A, genes involved in PUT synthesis displayed cell-type-specific expression: ODC1 was primarily expressed in B cells, and AGMAT in epithelial cells. Genes that inhibit PUT synthesis, such as *OAZ1*, were expressed mainly in myeloid and B cells, while NQO1 was highly expressed in endothelial and epithelial cells. Transport-related genes, such as *SLC7A1*, were mostly expressed in epithelial cells; *ATP13A3*, in myeloid cells; and *GPC1*, in fibroblasts mixed with glial cells. Subsequently, we investigated PUT-associated mechanisms in cell types with potential prognostic significance (Fig. 2B). Subpopulations within each major cell type were identified using non-NMF-based UMAP clustering (Fig. S3). MuSiC2 was then used to deconvolute the proportions of these subpopulations and assess their prognostic value. The dominant cell types in the MMR dataset were Endo_6, Epi_1, and B_6. In the TCGA dataset, Epi_6 and Endo_4 (Fig. 2C). Survival analysis revealed that Epi_6 was associated with a favorable prognosis, whereas Endo_5, Endo_6, FibroblastGlial_6, and Myeloid_5 were associated with poor prognosis in the MMR dataset. In the TCGA dataset, Epi_10 was linked to a better prognosis. At the same time, Endo_4, FibroblastGlial_3, FibroblastGlial_4, and T/NK/ILC_5 were associated with worse outcomes (Fig. 2D). Regarding GSVA scores for PUT metabolism, Endo_5, Endo_6, FibroblastGlial_6, and Myeloid_5 in the MMR dataset had higher extracellular than intracellular PUT concentrations, while Epi_6 showed the opposite pattern. Similarly, in the TCGA dataset, Endo_4, FibroblastGlial_3, FibroblastGlial_4, and T/NK/ILC_5 exhibited higher extracellular concentrations, whereas Epi_10 had lower extracellular than intracellular concentrations (Fig. 2E).

### PUT has a minimal impact on the malignant behavior of HCT-116 and RKO cells

**Figure 3:**
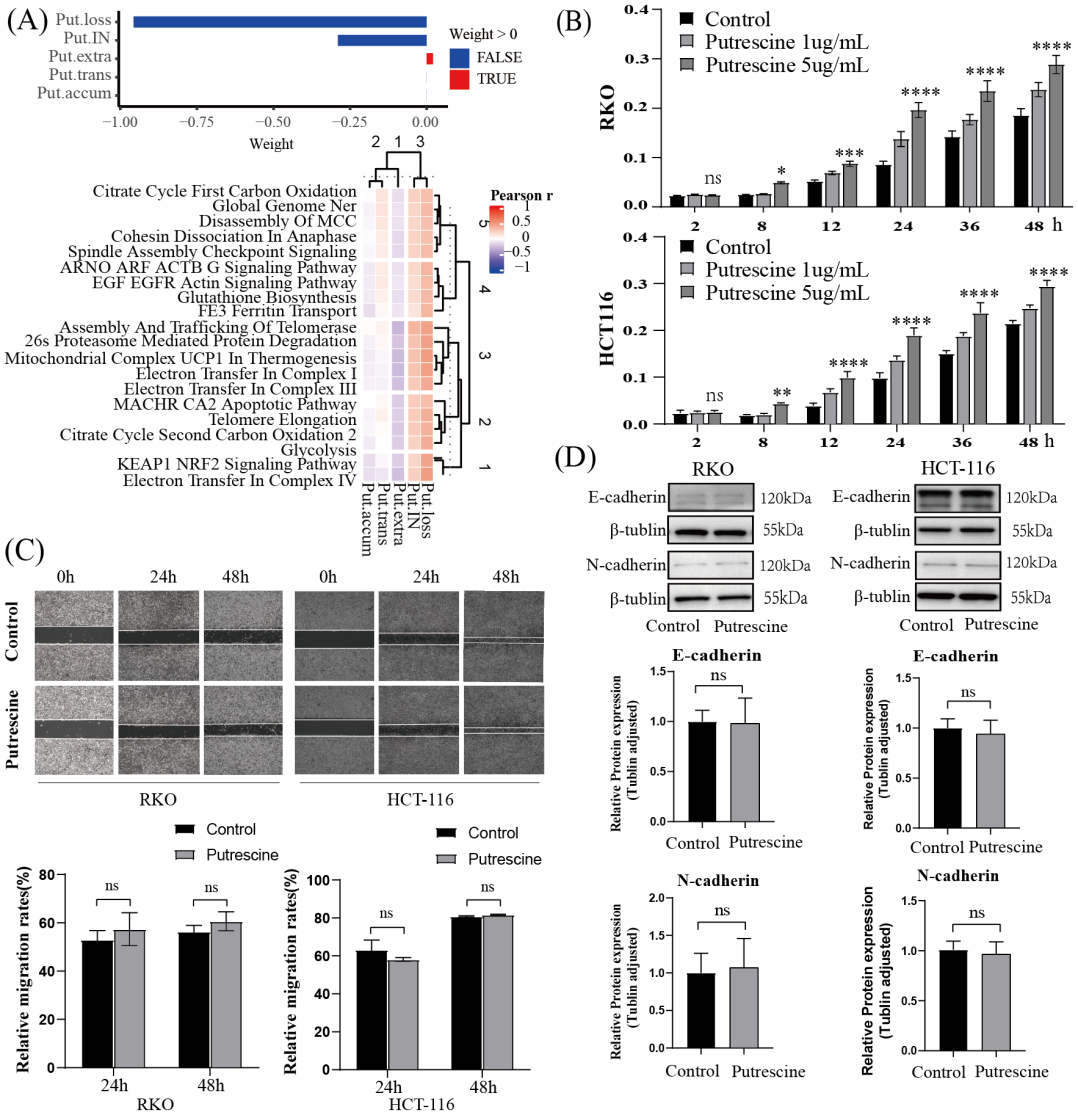
Impacts of supplemented putrescine on the malignant behavior of HCT-116 and RKO cells. (A) The weights of putrescine metabolisms in explaining the variance of KEGG pathways (top) based on CCA, and Pearson correlations between the putrescine metabolisms and top 20 associated KEGG pathways; (B) the absorbance of HCT-116 and RKO colon cancer cells stimulated by one µg/ml and five µg/ml putrescine at 490 nm at different time points was detected by MTT assay; (C) the cell migration of HCT-116 and RKO colon cancer cells stimulated by five µg/ml putrescine for 24 h and 48 h was detected by scratch test, statistical chart of the healed area of the scratch test were presented; (D) effects of five µg/ml putrescine on E-cadherin and N-cadherin in HCT-116 and RKO cells.

Figure 2 suggests that PUT metabolism has a negligible relationship with malignant epithelial cells. To further assess this, we employed CCA to evaluate the explanatory power of PUT metabolism concerning KEGG signaling pathways in epithelial cells. Among the metabolic features, PUT loss demonstrated the highest overall explanatory power for KEGG pathways. Among the top 20 pathways ranked by weight, the EGF-EGFR-Actin signaling pathway, spindle assembly checkpoint signaling, and cohesin dissociation pathways showed strong positive correlations with PUT biosynthesis and inhibition genes. However, these pathways showed weak correlations with PUT accumulation and extracellular levels (Fig. 3A). To assess the functional impact of PUT on CRC cells, we measured the proliferation of HCT-116 and RKO cells treated with supplemented PUT at final concentrations of one μg/mL and five μg/mL. An increase in cell number was observed in both cell lines with increasing concentrations of PUT (Fig. 3B). However, treatment with 5 μg/mL PUT did not significantly affect the migration of HCT-116 or RKO cells (Fig. 3C). Furthermore, the expression levels of N-cadherin and E-cadherin were not significantly altered in either cell line (Fig. 3D).

### Characterization of CXCR6^+^CD8^+^T cells in CRC

**Figure 4:**
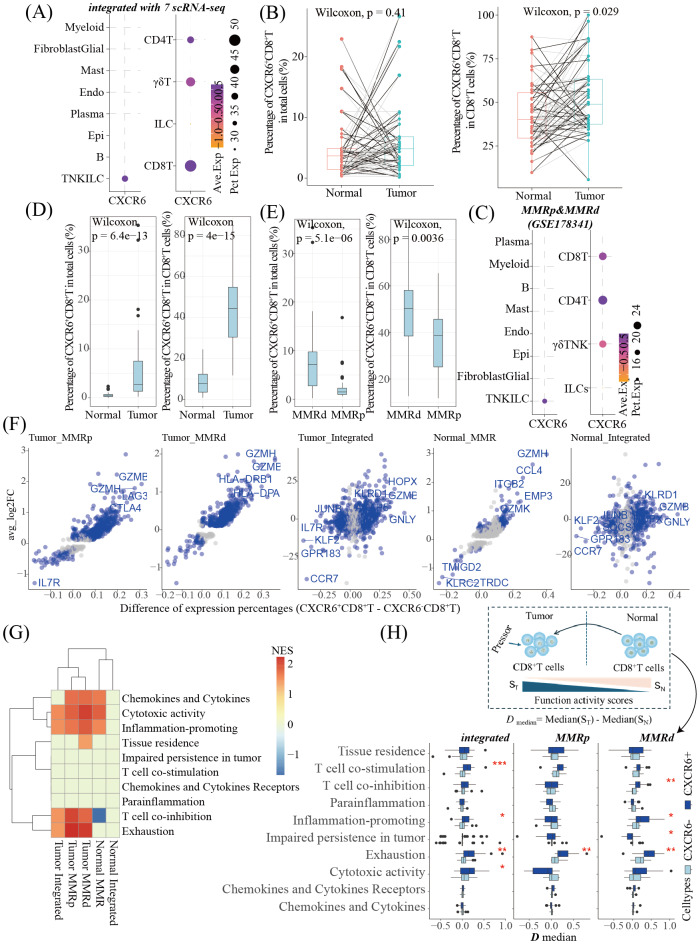
The percentages of CXCR6 expressing cells and CXCR6^+^CD8^+^T cells and the function of CXCR6^+^CD8^+^T cells were compared with CXCR6^−^CD8^+^T cells in the integrated scRNA-seq dataset and GSE178341. (A) The CXCR6 expressing cells in an integrated scRNA-seq of GSE132465, GSE144735, GSE161277, GSE166555, GSE188711, GSE200997, GSE221575; (B) the percentages of CXCR6^+^ CD8^+^ T cells in total cells and the CD8^+^ T cells from the integrated scRNA-seq dataset, the paired Wilcoxon test was employed to compare the percentages of tumor and normal tissues; (C) the CXCR6 expressing cells in GSE178341; (D–E) the percentages of CXCR6^+^ CD8^+^ T cells in total cells and the CD8^+^ T cells from the GSE178341 dataset, the Wilcoxon test was employed to compare the percentages in tumor and normal tissues (D) and the percentages in MMRd and MMRp (E); (F) the DEGs between CXCR6^+^ CD8^+^ T and CXCR6^−^ CD8^+^ T cells based on different subsets, the words ^+^ CD8^+^ T cells in responding to the pressor from suppressive tumor microenvironments. Only the patients with paired tumors and normal tissues, and paired CXCR6^+^ CD8^+^ T and CXCR6^−^CD8^+^ T cells were involved in Table S3.

In the integrated scRNA-seq dataset, CXCR6 was predominantly expressed in T/NK/ILC cells (Fig. 4A). These T/NK/ILC cells were further classified into CD4^+^ T cells, CD8^+^ T cells, γ*δ* T cells, NK cells, and ILCs (Fig. S4). CXCR6 expression was particularly high in CD8^+^ T cells (Fig. 4A). Although the overall percentage of CXCR6^+^CD8^+^ T cells among total cells did not differ between tumor and normal tissues, their proportion within the CD8^+^ T cell population was significantly higher in tumor tissues (Fig. 4B). In the GSE178341 dataset, which includes 285,551 cells from 100 samples across 62 subjects and compares MMRp and mismatch repair-deficient (MMRd) CRC, CXCR6 was also highly expressed in CD8^+^ T cells (Figs. S2, S5, Fig. 4C). Despite elevated proportions of CXCR6^+^CD8^+^ T cells in tumors relative to normal tissues (Fig. 4D), both the absolute and relative frequencies of these cells were lower in MMRp tumors than in MMRd tumors (Fig. 4E).

Next, we compared the functional profiles of CXCR6^+^CD8^+^ T cells with CXCR6^−^CD8^+^ T cells within the TME. Differential expression analysis between the two subsets in both datasets revealed elevated expression of cytotoxic genes—such as granzyme B (GZMB)—in the CXCR6^+^CD8^+^ T cell population (Fig. 4F). Functional activity was assessed in terms of chemokines and cytokines, their receptors, cytotoxicity, inflammation promotion, parainflammation, T cell co-inhibition, co-stimulation, exhaustion, impaired persistence within tumors, and tissue residency. As expected, CXCR6^+^CD8^+^ T cells exhibited higher cytotoxicity and pro-inflammatory activity than CXCR6^−^CD8^+^T cells in all tissues except for normal tissue in the integrated scRNA dataset. However, elevated levels of T cell co-inhibition and exhaustion were also observed across all tumor samples (Fig. 4G). Given the general dysfunction of T cells induced by the immunosuppressive TME, we examined the resilience of CXCR6^+^CD8^+^ T cells in this context. This was evaluated by comparing GSVA scores between tumor and normal tissues (*D*_*median*_, Fig. 4H). In the integrated scRNA dataset, CXCR6^+^CD8^+^ T cells exhibited higher *D*_*median*_ values for T cell co-stimulation, inflammation promotion, exhaustion, and cytotoxicity. In MMRp tumors, they showed higher *D*_*median*_ values for exhaustion. In contrast, MMRd tumors demonstrated increased values for co-inhibition, inflammation promotion, and exhaustion—but lower scores for impaired persistence in the TME.

**Figure 5:**
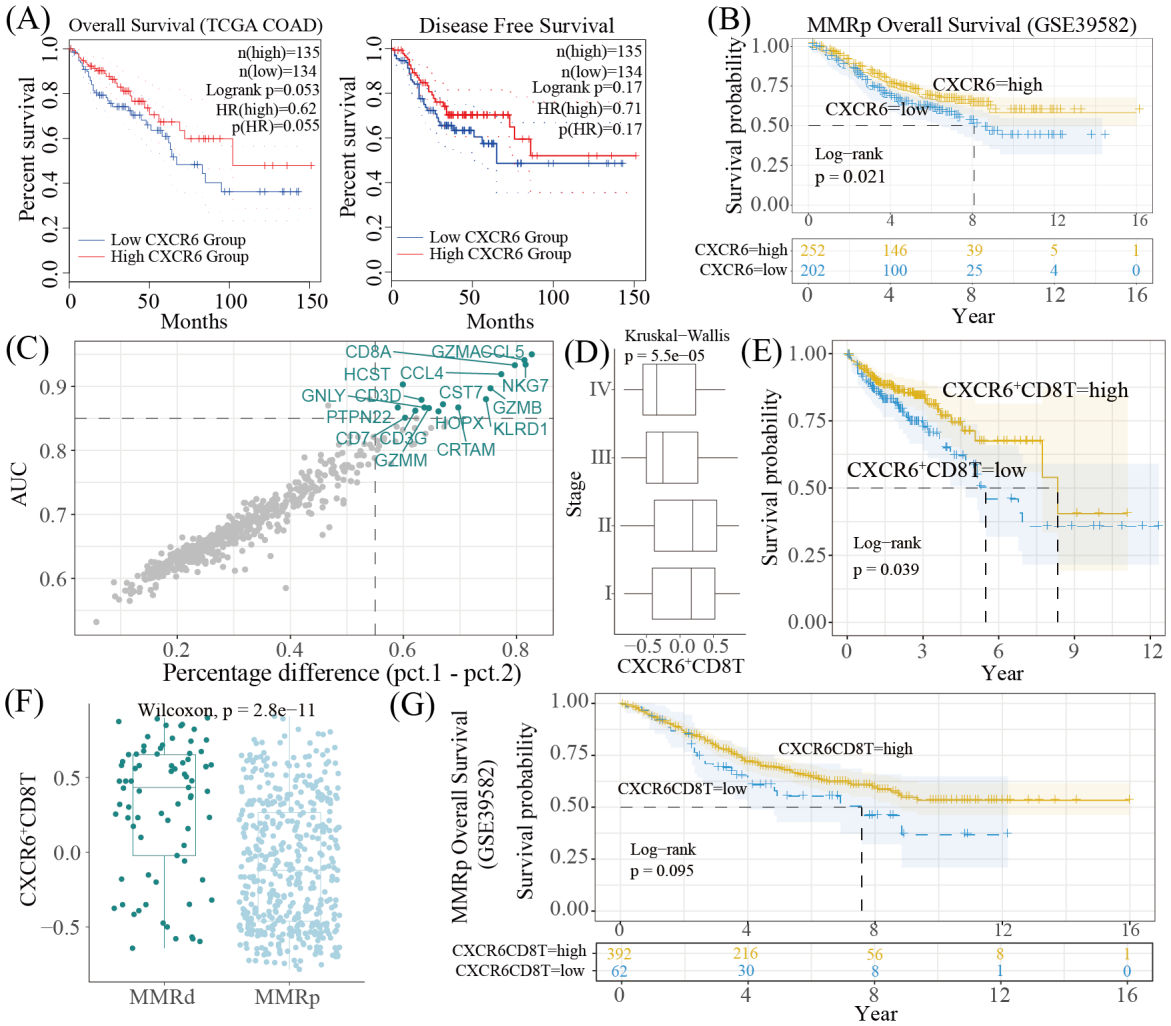
The prognostic abilities of CXCR6 and CXCR6^+^ CD8^+^ T cells in CRC. (A–B) KM survival curves comparing overall survival and disease-free survival between the CXCR6 high group and CXCR6 low group. Patients were stratified by the median cut-off, plots were generated using GEPIA2 based on TCGA-COAD cohort (A) and using survminer package based on GSE39582 dataset (B); (C) the marker genes for CXCR6^+^ CD8^+^ T cells; (D) boxplot comparing the GSVA scores of the CXCR6^+^ CD8^+^ T cells in different stages based on TCGACOAD dataset; (E, G) KM survival curves comparing overall survival between the CXCR6^+^ CD8^+^ T high group and CXCR6^+^ CD8^+^ T low group. Patients were stratified by the median cut-off, plots were generated based on TCGA-COAD cohort (E) and GSE39582 dataset (G); (F) boxplot comparing the GSVA scores of the CXCR6^+^ CD8^+^T cells in MMRd patients and MMRp patients.

We then assessed the prognostic significance of CXCR6 expression and CXCR6^+^CD8^+^ T cell infiltration in CRC. As anticipated, higher CXCR6 expression correlated with improved prognosis in both the TCGA-COAD dataset (Fig. 5A) and the MMRp cohort (Fig. 5B). To evaluate the prognostic value of CXCR6^+^CD8^+^ T cells, marker genes for this population were identified using ROC analysis based on the integrated scRNA dataset, which provided superior data quality compared to GSE178341. As shown in Fig. 5C, in addition to *CD3D*, *CD3E*, *CD8A*, *CD8B*, and *CXCR6*, genes with an AUC > 0.85 and a percentage difference >0.55 were selected as markers. GSVA scores calculated from the TCGA-COAD dataset showed a decline in CXCR6^+^CD8^+^ T cell infiltration from stages I/II to stages III/IV (Fig. 5D). Higher GSVA scores of these cells were significantly associated with better prognosis in this cohort (Fig. 5E). In the GSE39582 dataset, their GSVA scores were higher in MMRd tumors compared to MMRp tumors (Fig. 5F). Furthermore, among MMRp patients, elevated GSVA scores for CXCR6^+^CD8^+^ T cells showed a trend toward improved prognosis. However, the *p*-value was 0.095 (Fig. 5G).

### PUT impairs the surveillance function of CXCR6^+^CD8^+^T cells

**Figure 6:**
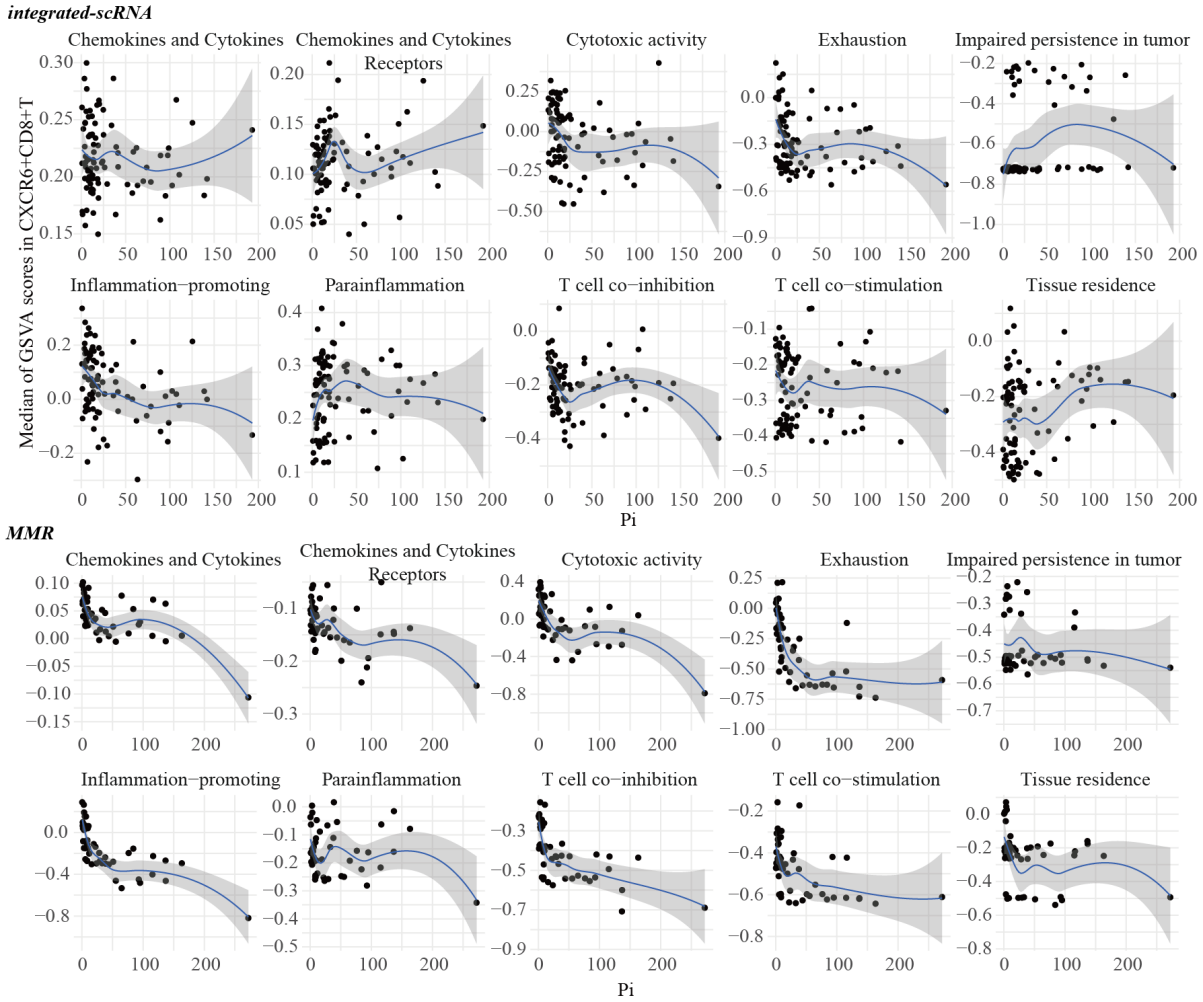
Associations between the putrescine accumulation and the functions of the CXCR6^+^ CD8^+^ T cells. The associations between the GSVA scores of functional gene sets and the *P* values in integrated-scRNA and MMR.

The S_PA_ score, which quantifies intracellular PUT accumulation, was highest in T/NK/ILC, epithelial cells, and mast cells, and lowest in myeloid cells (Fig. S6). However, most CXCR6^+^CD8^+^ T cells had S_PA_ values of zero, precluding any meaningful correlation with functional gene sets (Figs. S7–S8). We therefore focused on the Pi score, which estimates the pericellular accumulation of PUT surrounding CXCR6^+^CD8^+^ T cells. Strikingly, the Pi score negatively correlated with cytotoxic activity and inflammation-promoting functions in CXCR6^+^CD8^+^ T cells across both scRNA-seq datasets. A negative correlation was also observed between Pi values and exhaustion scores (Fig. 6).

**Figure 7:**
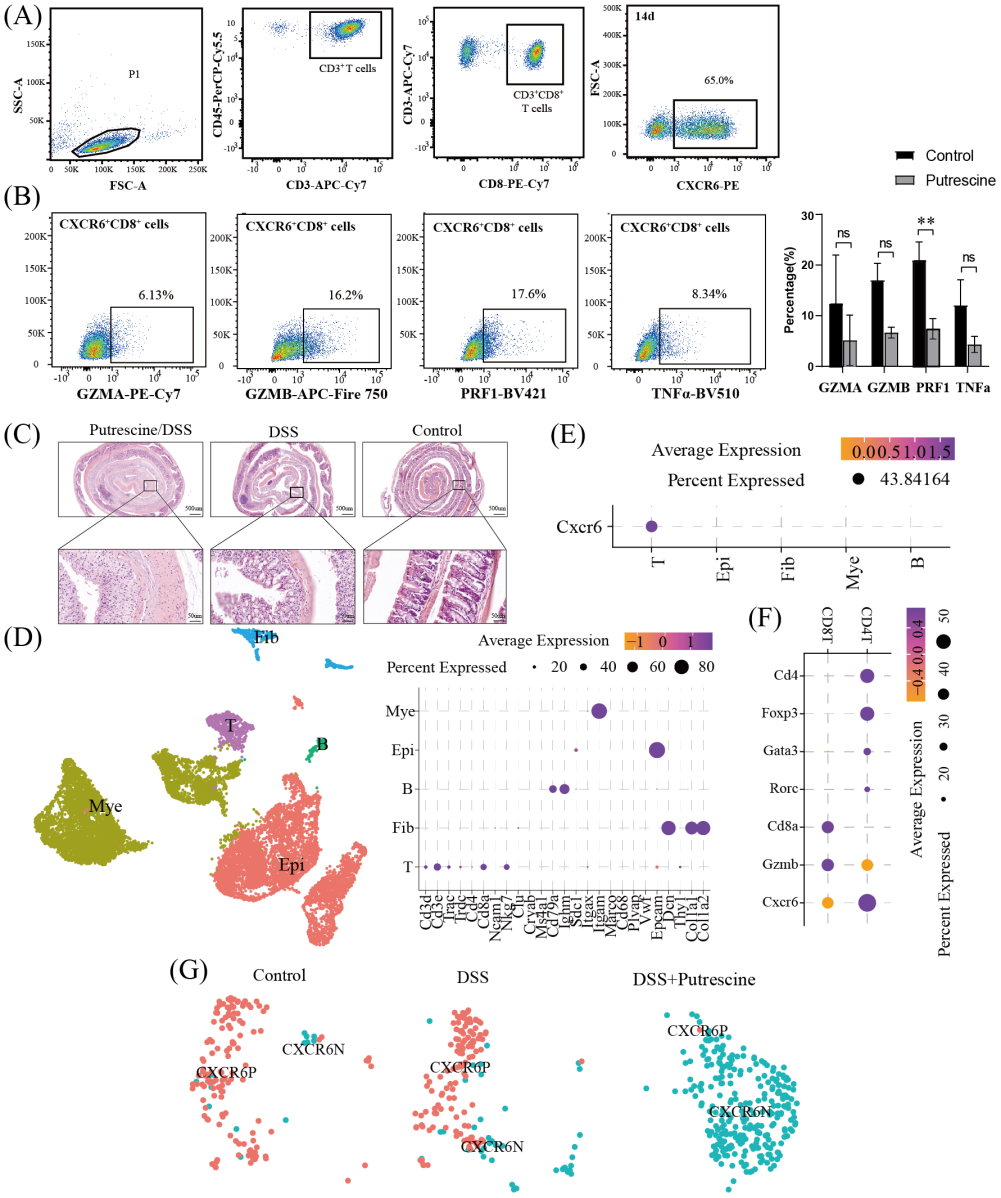
Effects of putrescine on CXCR6^+^ CD8^+^ T cell cytotoxicity and on CXCR6^+^ CD8^+^ T cells in DSS-induced colitis. (A) Flow cytometry gating strategy for identifying CXCR6^+^CD8^+^ T cells. Dead cells were excluded using Zombie NIR viability dye prior to CXCR6^+^CD8^+^ T-cell analysis. (B) Representative flow cytometry plots showing the expression percentages of GZMA, GZMB, PRF1, and TNF-α, with bar plots comparing expression levels between the putrescine-treated and control group; (C) hematoxylin and eosin (HE) staining the colon in the control, DSS, and DSS/Putrescine groups; (D) dimplot and dotter plot illustrating the assignments of all cells and the expression of classical marker genes; (E–F) dotter plot illustrating the expression of CXCR6 on the broad cell types (E) and on the CD4^+^ T and CD8 ^+^ T cells (F); (G) separated dimplots illustrating the distribution of CXCR6^+^ CD8^+^ T cells in the three groups.

**Figure 8:**
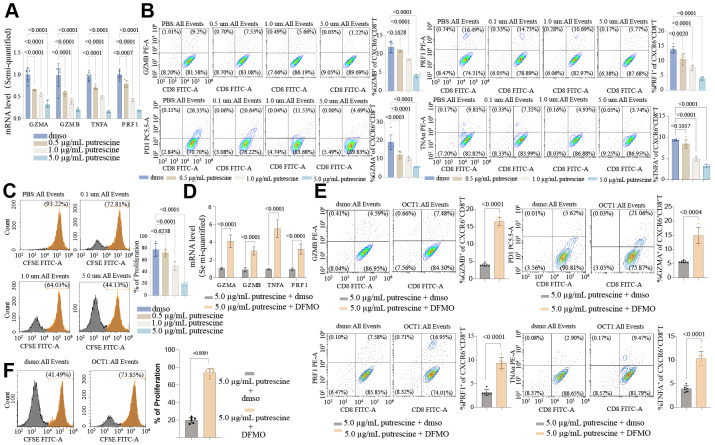
Putrescine suppresses the cytotoxicity and proliferation of CXCR6^+^ CD8^+^ T cells through an ODC1-dependent mechanism. (A) CXCR6^+^CD8^+^T Cells were cultured *in vitro* with zero, one, or five µg/mL putrescine for 48 h, the mRNA of PRF1, GZMB, TNFA and GZMA. (B) Representative flow cytometry plots and quantitative analysis showing the expression and secretion levels of PRF1, GZMB, TNFA and GZMA in CXCR6^+^CD8^+^ T cells after putrescine treatment. (C) Cell proliferation assessed by CFSE dilution assay demonstrating that putrescine exposure significantly limits the proliferation of CXCR6^+^CD8^+^ T cells in a dose-dependent manner. (D) The rescue experiment using Difluoromethylornithine (DFMO), a specific inhibitor of ODC1, the rate-limiting enzyme initiating polyamine synthesis. mRNA of PRF1, GZMB, TNFA and GZMA. (E) Flow cytometry plots and quantification demonstrating that DFMO co-treatment reverses the inhibitory effects of putrescine on PRF1, GZMB, and IL-2 expression. (F) Cell proliferation assessed by CFSE dilution assay demonstrating that DFMO treatment restores the proliferation and cytotoxic potential of CXCR6^+^CD8^+^ T cells suppressed by extracellular putrescine. Data are presented as mean ± SD from three independent biological replicates. Statistical analysis was performed using one-way ANOVA followed by Tukey’s *post hoc* test; *p* < 0.05 was considered statistically significant.

To determine whether direct PUT impairs cytotoxic activity, we induced CXCR6^+^CD8^+^ T cells *in vitro*. By day 14, over 60% of CD8^+^ T cells expressed CXCR6^+^CD8^+^T cells (Fig. 7A). PUT treatment significantly reduced the expression of cytotoxicity-related markers in these cells (Fig. 7B). To further investigate the impact of PUT on immune surveillance, we employed a DSS-induced colitis model. As shown in Figs. 7C and 7D, DSS-fed mice exhibited disrupted colonic architecture and reduced goblet cell numbers compared to controls. The colonic injury was more severe in mice administered DSS and PUT, with marked mucosal congestion, inflammatory infiltration, decreased goblet cell counts, and muscular edema. These findings indicate that PUT significantly exacerbates colitis-associated inflammation. All cells were subsequently classified into myeloid cells, epithelial cells, B cells, fibroblasts, and T cells based on classical marker gene expression (Fig. 7D). CXCR6 was exclusively expressed in T cells (Fig. 7E) and was highly expressed in both CD8^+^ T cells and regulatory T cells (CD4^+^Foxp3^+^ Tregs) (Fig. 7F). Notably, Cxcr6^+^Cd8^+^ T cells were completely absent in the PUT-treated group (Fig. 7G). To investigate the role of PUT in modulating the cytotoxicity of CXCR6^+^CD8 T cells, CXCR6^+^CD8 T cells were isolated from the spleens of mice and treated with 0, 1, or 5 µg/mL PUT. Similar to *in vivo* observations, PUT treatment significantly reduced the expression and secretion of GZMB, perforin 1 (PRF1), IL-2, and GZMB (Figs. 8A, 8B). PUT treatment also significantly inhibited the proliferation of CXCR6^+^CD8 T cells (Fig. 8C). To confirm the involvement of PUT in CXCR6^+^CD8 T-cell dysfunction, difluoromethylornithine (DFMO), a specific inhibitor of ornithine decarboxylase 1 (ODC1)—the enzyme initiating polyamine biosynthesis—was added to cultures treated with five µg/mL PUT. DFMO treatment reversed the inhibitory effects of PUT on the cytotoxicity of CXCR6^+^CD8 T cells (Figs. 8D–8F).

## Discussion

In this study, we first investigated the prognostic relevance of PUT metabolism in CRC. Based on previous studies (Liu et al., 2022; Meng et al., 2022), we initially hypothesized that higher levels of PUT biosynthesis—mediated by ODC1 and AGMAT—would be associated with a worse prognosis. However, we observed the opposite association in two independent cohorts (Fig. 1). One possible explanation is that ODC1 and AGMAT play significant positive roles in both tumor and anti-tumor immune cells, with their expression markedly higher in the latter. Using integrated scRNA-seq datasets comprising 221 samples from 131 subjects, we found that ODC1 and AGMAT were highly expressed in B cells and epithelial cells (Fig. 2). However, when we applied non-NMF-based UMAP and MuSiC2 deconvolution methods, the GSVA scores for PUT biosynthesis were found to be elevated in four epithelial subpopulations and one B cell subpopulation in the MMR scRNA-seq dataset, and in two epithelial and one B cell subpopulation in the integrated dataset. Notably, these epithelial subpopulations were associated with better prognosis, whereas the B-cell subpopulation did not demonstrate significant prognostic value. In contrast, genes involved in biosynthesis inhibition and polyamine transport were associated with poorer outcomes (Fig. 1). Furthermore, in the TCGA-COAD cohort, the Epi_7 subpopulation, which had the best prognosis, exhibited the lowest scores for biosynthesis inhibition. Conversely, endothelial and fibroblast subpopulations, which displayed relatively higher GSVA scores for biosynthesis inhibition and transport, were linked to poor prognosis (Fig. 2). Since genes inhibiting biosynthesis are also involved in polyamine transport (Holbert et al., 2022), these results suggest that PUT transport, rather than biosynthesis, drives CRC progression. This finding aligns with previous reports showing that combining ODC1 inhibitors (*e.g.*, DFMO) with polyamine transport inhibitors (*e.g.*, AMXT 1501) suppresses tumor growth *in vivo* in models of melanoma, colon cancer, and mammary adenocarcinoma (Alexander et al., 2020; Gitto et al., 2018).

Our previous study demonstrated that supplementation with PUT could enhance EMT in gastric cancer cells (Bi et al., 2024). However, in the present study, although extracellular PUT promoted CRC tumor cell proliferation, it did not affect the EMT process (Fig. 3). Moreover, polyamine-blocking therapy is ineffective under immunodeficient conditions, underscoring the critical role of the immune system in mediating its therapeutic effects (Hayes, Burns & Gilmour, 2014). Accordingly, we focused on uncovering the immunological mechanisms underlying the association between poor prognosis and enhanced PUT transport. Previous research has highlighted the central role of T cells in anti-tumor immunity. In particular, CXCR6 is crucial for the anti-tumor activity of cytotoxic T cells. We first examined CXCR6 expression in CRC using integrative multi-omics analyses. CXCR6 is selectively expressed on the surface of T and NK lymphocytes within the TME of solid tumors, especially on CD8+ T cells, and is absent in B cells and non-lymphoid immune cells (Di Pilato et al., 2021; Kim & Kim, 2019; Wang et al., 2021). However, some studies have reported that CXCR6 can also be expressed by solid tumor cells and is associated with metastasis and tumor progression (Korbecki et al., 2021). High CXCR6 expression in liver cancer has been linked to increased aggressiveness of hepatocellular carcinoma, poor prognosis, and serves as a predictor of recurrence (Gao et al., 2012). In our analysis of integrated scRNA-seq datasets, CXCR6 expression in CRC was predominantly observed in T cells (Fig. 1). We hypothesized that CXCR6^+^CD8^+^ T cells possess a high immune surveillance capacity in CRC. To evaluate their functional characteristics, particularly those related to surveillance ability, we defined an updated gene signature comprising 10 functional features. These included chemokine and cytokine receptor expression, cytotoxic activity, inflammation promotion, T cell co-stimulation, and tissue residency—each enhancing surveillance. Conversely, T cell co-inhibition, exhaustion, and impaired persistence in tumors were associated with diminished surveillance or anti-tumor activity. The feature of tissue residency was newly introduced and emphasized in this study. Tissue-resident memory T cells (Trm) are a subset of T cells that reside within tissues and are characterized by the expression of CD69 and CD103a and the absence of S1PR1 and CCR7 (Crowl et al., 2022). Numerous studies have highlighted the importance of Trm cells in anti-tumor immunity due to their abundance, phenotypic heterogeneity, and unique ability to mount rapid immune responses (Anadon et al., 2022; Yang & Kallies, 2021; Yenyuwadee et al., 2022). CXCR6 has also been identified as a marker of Trm cells in certain cancers (Mabrouk et al., 2022). In the current study, consistent with previous findings, we observed elevated tissue residency scores for CXCR6^+^CD8^+^ T cells within MMRd tumor tissues based on the GSE178341 dataset.

As expected, we observed a higher percentage of CXCR6^+^CD8^+^ T cells in tumor tissue compared to normal tissue (Fig. 4). These cells also showed elevated expression of cytotoxic genes, along with enhanced cytotoxic and inflammation-promoting activities, in tumor tissues compared to normal tissues (Fig. 4). Given the known immunosuppressive TME that impairs T cell function we questioned whether CXCR6^+^CD8^+^ T cells are more resistant to such suppression and more responsive to CRC tumors. To evaluate this, we considered the relative increase in cytotoxic activity between tumor and normal tissues. If the rise in a given functional activity (*e.g.*, cytotoxicity) is greater in one cell type (cell A) than in another (cell B), cell A can be interpreted as more responsive. In this study, we observed a greater increase in cytotoxic activity in CXCR6^+^CD8^+^ T cells compared to CXCR6^−^CD8^+^T cells, even in MMRp CRC, which typically has a lower mutational burden and weaker immune responsiveness than MMRd CRC (Pelka et al., 2021). Therefore, it is not surprising that CXCR6^+^CD8^+^ T cells were associated with a better prognosis (Fig. 5). However, we also observed that CXCR6^+^CD8^+^ T cells exhibited higher levels of exhaustion than their CXCR6^−^CD8^+^T cells. Previous studies have shown that CXCR6 is essential for the survival, expansion, and maintenance of cytotoxic T lymphocyte function within the TME (Di Pilato et al., 2021; Wang et al., 2021), which may appear to contradict our findings. Furthermore, as CXCR6^+^CD8^+^ T cells represent a key subset responsive to immune checkpoint blockade (Wang et al., 2021), and given that reversing CD8^+^ T cell exhaustion is a major goal in immunotherapy (Zander & Cui, 2023), it becomes imperative to elucidate further the role of CXCR6^+^CD8^+^ T cells in CRC.

Given their critical role in anti-tumor immunity, we next investigated the association between PUT metabolism and the functional activity of CXCR6^+^CD8^+^ T cells. Although previous research has mainly focused on the effects of putrescine on CD4+ T cells—particularly Th17 cells, given their relevance in autoimmunity (Wagner et al., 2021)—there has been limited investigation of its impact on CD8^+^ T cells. In this study, we demonstrated that PUT impairs the cytotoxic function of CXCR6^+^CD8^+^ T cells. We developed SPA and Pi indices to assess PUT accumulation, not biosynthesis alone. We found that the *P* values for other cell types showed negative correlations with the functional activities of CXCR6^+^CD8^+^ T cells. This suggests that local PUT accumulation has a more detrimental effect on these cells than its biosynthesis per se. Furthermore, supplementation of PUT during *in vitro* induction of CXCR6^+^CD8^+^ T cells confirmed its negative impact on their cytotoxic activity (Fig. 7). Ulcerative colitis (UC), a major risk factor for CRC due to its association with colitis-associated cancer (CAC), further motivated our investigation (Shah & Itzkowitz, 2022). The incidence of CRC in Asian patients with UC has been reported at 0.02%, 4.81%, and 13.91% after 10, 20, and 30 years of disease duration, respectively (Bopanna et al., 2017). Impaired immune surveillance in UC is a key contributor to CAC development. We, therefore, employed a DSS-induced UC model to evaluate the effects of PUT on CXC6^+^CD8^+^ T cells. PUT administration exacerbated colon inflammation, consistent with previous findings (Grosheva et al., 2020). Using scRNA-seq, we confirmed that CXCR6 was predominantly expressed by T cells, particularly CD8^+^ T cells (Fig. 7). Further analysis revealed that PUT administration led to a depletion of CXCR6^+^CD8^+^ T cells during DSS-induced UC. This provides a potential mechanism linking PUT accumulation to CAC development. However, a noteworthy contradiction emerged: although putrescine aggravated inflammation, it also impaired the inflammation-promoting functions of CXCR6^+^CD8^+^ T cells. This suggests that putrescine may exert effects beyond T cells.

The *in vitro* experiments provide mechanistic insights into the immunosuppressive effects of PUT. Treatment of mouse splenic CXCR6^+^CD8^+^ T cells with one or five µg/mL PUT significantly reduced the expression and secretion of cytotoxic effectors (GZMB, PRF1) and IL-2 while inhibiting proliferation (Figs. 8A–8C). Co-treatment with DFMO, an ODC1 inhibitor, restored cytotoxic activity and proliferation (Figs. 8D–8F), confirming that PUT impairs T-cell receptor signaling and metabolic fitness *via* an ODC1-dependent pathway. These findings establish a causal link between extracellular PUT accumulation and CXCR6^+^CD8^+^ T-cell dysfunction, extending multi-omics associations.

They also highlight polyamine metabolism as a potential therapeutic target in CRC. DFMO and polyamine transport inhibitors, which have already been tested in early-phase oncology trials, may enhance CXCR6^+^CD8^+^ T-cell function and synergize with immune checkpoint blockade. Stratifying patients based on PUT transport gene expression or polyamine index values could identify individuals most likely to benefit from polyamine-targeted therapies, enabling precision immunotherapy. Although these analyses were validated across multiple public datasets, independent prospective cohorts are required to confirm these findings at both transcriptomic and functional levels. Future work will involve analyzing patient samples collected at collaborating clinical centers to validate the identified prognostic and immunological associations prospectively. A limitation of this study is the absence of direct *in vivo* depletion experiments confirming the functional necessity of CXCR6^+^CD8^+^ T cells in CRC. Future research employing CXCR6-targeted depletion or conditional knockout models will be essential to establish causality. Additional pathway-level studies, including metabolic tracing and signal transducer and activator of transcription 3/mechanistic target of rapamycin complex 1 activity assays, may further clarify how extracellular PUT modulates CD8^+^ T-cell cytotoxicity. Although our integrative analyses demonstrate a strong correlation between extracellular PUT accumulation and reduced CXCR6^+^CD8^+^ T-cell cytotoxicity, these findings remain associative. Combining polyamine-transport blockade with CXCR6^+^CD8^+^ T-cell depletion will be necessary to establish direct causality and elucidate the molecular checkpoints through which PUT modulates antitumor immunity. While our study was initially based on multi-omics integration, the inclusion of *in vitro* and *in vivo* validation experiments substantiates the computational predictions. Collectively, these findings indicate that extracellular PUT accumulation functionally suppresses CXCR6^+^CD8^+^ T-cell cytotoxicity, although further mechanistic validation is warranted to confirm the signaling pathways involved.

Although state-of-the-art integration algorithms were applied to minimize batch effects and technical heterogeneity, residual confounding arising from differences in sequencing platforms, sample preparation, or patient demographics cannot be entirely excluded. Future studies utilizing uniformly processed, prospectively collected datasets will be essential to validate the observed associations under fully standardized conditions.

### Conclusion

This study demonstrates that PUT biosynthesis in tumor tissue is not associated with poor prognosis in CRC. Instead, polyamine transport and extracellular PUT play more significant roles in CRC progression. The underlying mechanism was that PUT disrupted the surveillance of cytotoxic T cells, particularly CXCR6+CD8+ T cells, on tumor cells rather than promoting extracellular PUT to alter the malignant behavior of tumor cells. Nonetheless, this study has several limitations. Direct evidence from CXCR6^+^CD8^+^ T cell-depletion models in mice is lacking, making it difficult to determine their role in CRC development conclusively. Additionally, the molecular mechanisms through which PUT inhibits the cytotoxic function of CD8^+^ T cells remain unclear. Future research should address these gaps. Future experiments will employ conditional mouse models, such as Odc1^*fl*/*fl*^ or Srm^*fl*/*fl*^ crossed with CD8a-Cre and Cxcr6-Cre lines, to specifically manipulate PUT synthesis or uptake in CD8^+^ T cells. *In vivo* administration of DFMO or polyamine transport inhibitors will further confirm the direct immunomodulatory effects of extracellular PUT on antitumor immunity.

## Supplemental Information

10.7717/peerj.20663/supp-1 Supplemental Information 1 Cell-type annotation in the integrated single-cell RNA-seq dataset UMAP visualization of the integrated scRNA-seq dataset (seven cohorts: GSE132465, GSE144735, GSE161277, GSE166555, GSE188711, GSE200997, and GSE221575) after Harmony-based batch correction. Major cell types were identified using canonical marker genes: epithelial cells (EPCAM, KRT18), endothelial cells (PECAM1, VWF), T/NK/ILC cells (CD3D, NKG7), B cells (MS4A1), plasma cells (MZB1), myeloid cells (CD68, LYZ), mast cells (TPSAB1), and fibroblast/glial cells (COL1A1, GFAP).

10.7717/peerj.20663/supp-2 Supplemental Information 2 Cell-type annotation and validation in the GSE178341 (MMR) single-cell dataset UMAP plots of 285,551 cells from 100 CRC samples (62 patients) classified into eight major lineages based on marker expression. Panels show representative feature plots for EPCAM, PECAM1, CD3D, MS4A1, LYZ, and COL1A1. The distribution of mismatch repair–proficient (MMRp) and –deficient (MMRd) tumors across major cell types is also shown.

10.7717/peerj.20663/supp-3 Supplemental Information 3 Subclustering of epithelial, endothelial, myeloid, fibroblast, and lymphoid compartments Non-negative matrix factorization (NMF)–based UMAP clustering within each major lineage identifies transcriptionally distinct subpopulations (*e.g.*, Epi_1–Epi_10, Endo_1–Endo_6, Myeloid_1–Myeloid_5). Representative marker genes for each subpopulation are shown in heatmaps.

10.7717/peerj.20663/supp-4 Supplemental Information 4 Classification of T/NK/ILC compartments into functional subsets UMAP visualization and feature plots showing expression of CD4, CD8A, TRDC, NCR1, and IL7R, delineating CD4^+^ T cells, CD8^+^ T cells, γ*δ* T cells, NK cells, and innate lymphoid cells (ILCs). CXCR6 expression is primarily enriched in the CD8^+^ T-cell subset.

10.7717/peerj.20663/supp-5 Supplemental Information 5 CXCR6 expression patterns in MMRp and MMRd colorectal cancer Violin and feature plots showing CXCR6 expression across tumor and normal tissues in MMRp and MMRd subgroups from GSE178341. The proportion of CXCR6^+^CD8^+^ T cells is elevated in tumors compared to adjacent normal tissue and higher in MMRd relative to MMRp tumors.

10.7717/peerj.20663/supp-6 Supplemental Information 6 Distribution of intracellular and extracellular putrescine accumulation across cell types Boxplots showing SPA (intracellular accumulation) and Pi (pericellular accumulation) scores for putrescine across eight major cell types in the integrated scRNA-seq dataset. T/NK/ILC cells, epithelial cells, and mast cells exhibit the highest SPA values, while myeloid cells show the lowest.

10.7717/peerj.20663/supp-7 Supplemental Information 7 Correlation between pericellular putrescine (Pi) scores and T-cell functional gene sets Spearman correlation analysis between Pi scores and GSVA enrichment scores of cytotoxicity, inflammation promotion, exhaustion, and co-inhibition pathways in CXCR6^+^CD8^+^ T cells. Higher pericellular putrescine levels negatively correlate with cytotoxic and inflammatory signatures, supporting functional suppression of CXCR6^+^CD8^+^ T cells.

10.7717/peerj.20663/supp-8 Supplemental Information 8 Correlation between different functional gene sets in MMR and median SPA values in CXCR6^+^CD8^+^T cells

10.7717/peerj.20663/supp-9 Supplemental Information 9 Putrescine metabolism gene setPutrescine metabolism gene set

10.7717/peerj.20663/supp-10 Supplemental Information 10 T cell functional gene set

10.7717/peerj.20663/supp-11 Supplemental Information 11 Patients whose tumors were paired with normal tissue, and patients whose CXCR6+CD8+T and CXCR6-CD8+T cells were paired

10.7717/peerj.20663/supp-12 Supplemental Information 12 Raw flow cytometry data

10.7717/peerj.20663/supp-13 Supplemental Information 13 Original WB

10.7717/peerj.20663/supp-14 Supplemental Information 14 Author Checklist - Full

## Additional Information and Declarations

### Data Availability

The following information was supplied regarding data availability: The sequencing data is available at Zenodo: - Tan, Z. (2025). R.Data [Data set]. Zenodo. https://doi.org/10.5281/zenodo.14997262
- Tan, Z. (2025). R.Data2 [Data set]. Zenodo. https://doi.org/10.5281/zenodo.14997489
The original pictures and the processed Single cell RNA sequencing data are available at figshare: - Zhang, Yuqiang (2025). Multiomics analysis on polyamine metabolisms in colorectal cancer indicates important contribution of impaired anti-tumor activities of CXCR6+CD8+T cells by the extracellular putrescine. figshare. Dataset. https://doi.org/10.6084/m9.figshare.28816250.v4. The processed data are available at OMIX, China National Center for Bioinformation/Beijing Institute of Genomics, Chinese Academy of Sciences: PRJCA036189. The single cell RNA sequencing data are available at the National Genomics Data Center: CRA024755.
