# Supplementary figures and images for: Multiomics analysis of polyamine metabolism in colorectal cancer, highlighting the key role of extracellular putrescine in impairing CXCR6+CD8+ T cell anti-tumor activity
Source: PeerJ. 2026 Feb 12;14:e20663. doi: 10.7717/peerj.20663 (PMC12906710; doi:10.7717/peerj.20663)

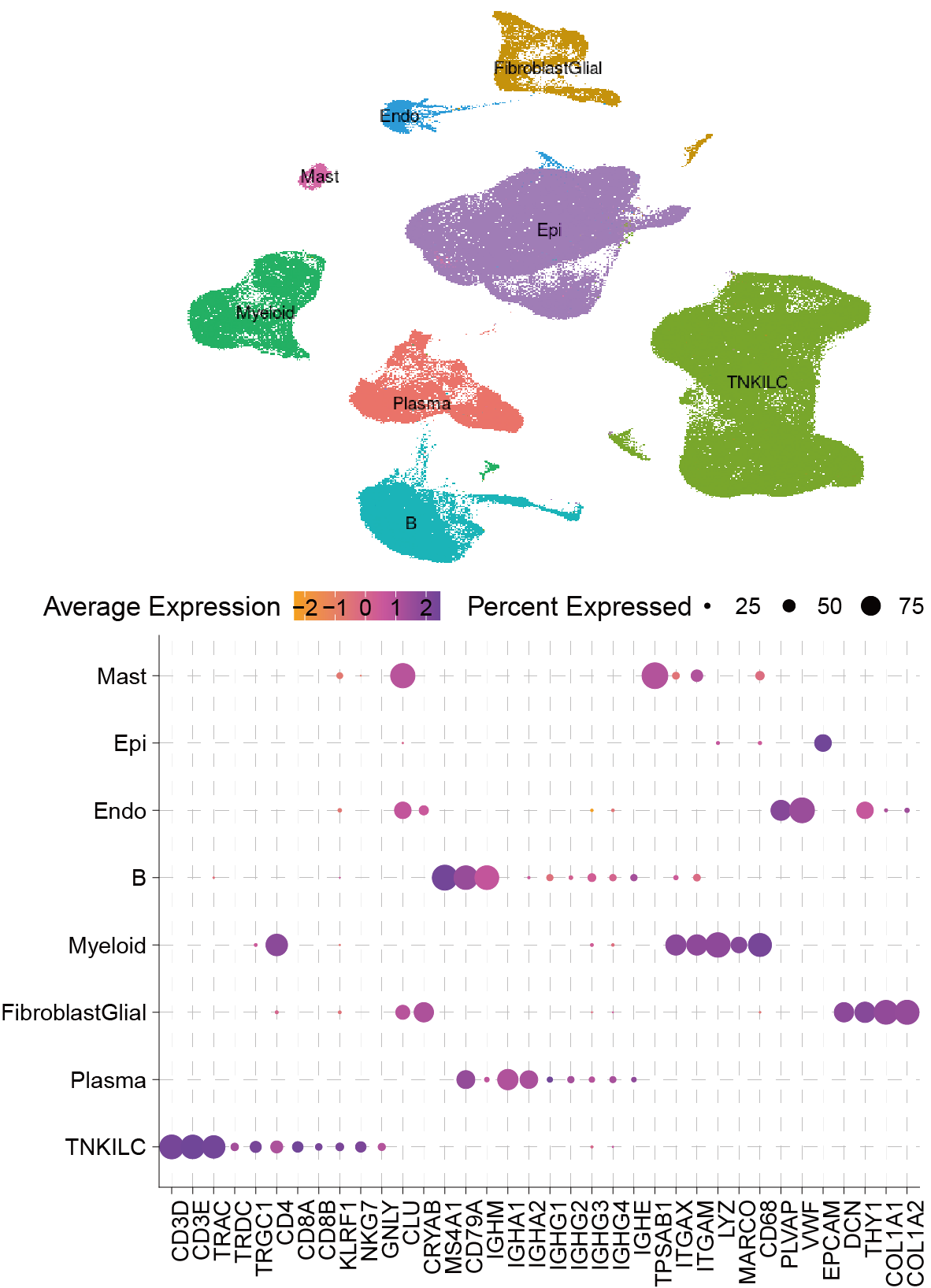

Supplement: Supplemental Information 1 — UMAP visualization of the integrated scRNA-seq dataset (seven cohorts: GSE132465, GSE144735, GSE161277, GSE166555, GSE188711, GSE200997, and GSE221575) after Harmony-based batch correction. Major cell types were identified using canonical marker genes: epithelial cells (EPCAM, KRT18), endothelial cells (PECAM1, VWF), T/NK/ILC cells (CD3D, NKG7), B cells (MS4A1), plasma cells (MZB1), myeloid cells (CD68, LYZ), mast cells (TPSAB1), and fibroblast/glial cells (COL1A1, GFAP). [file peerj-14-20663-s001.png]

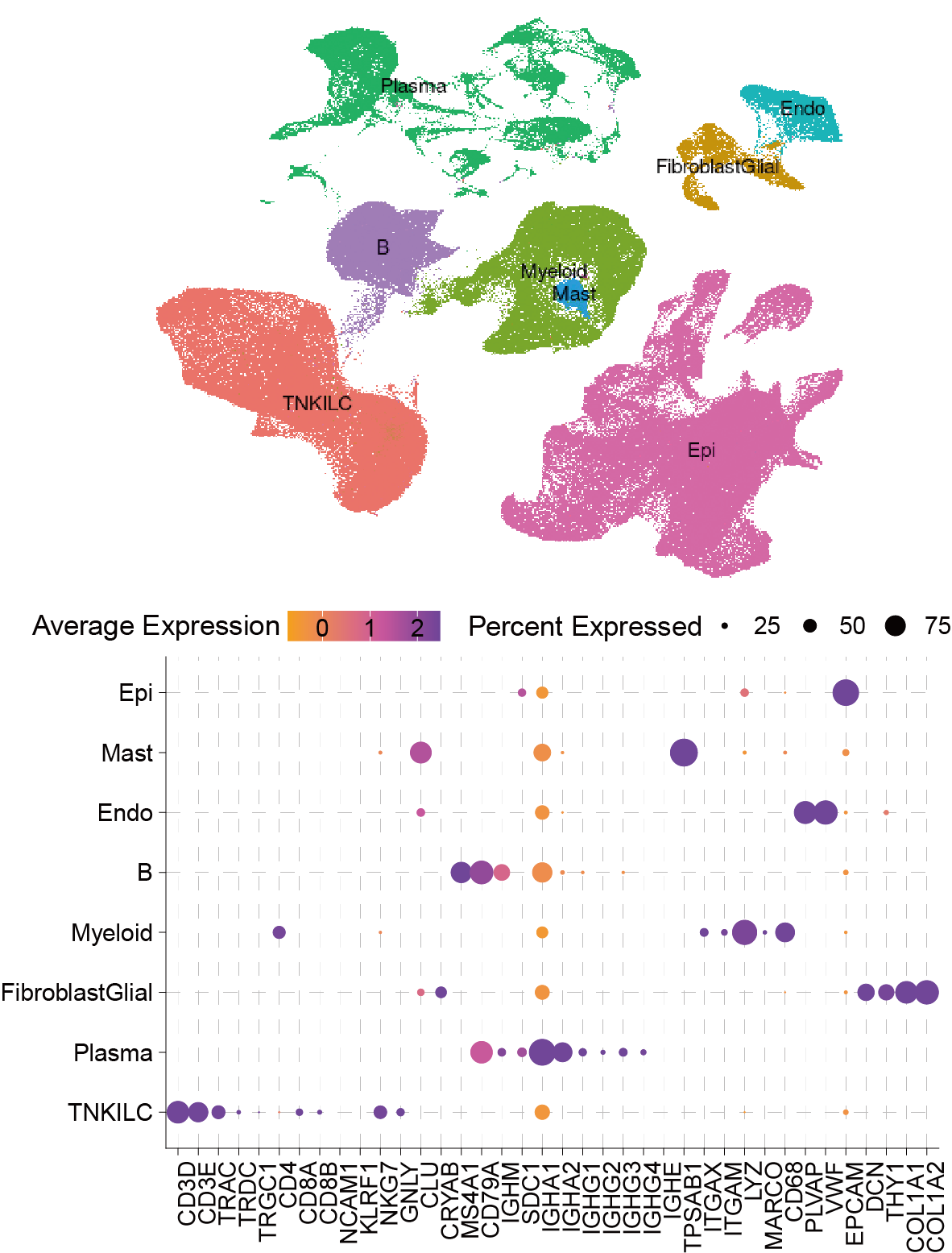

Supplement: Supplemental Information 2 — UMAP plots of 285,551 cells from 100 CRC samples (62 patients) classified into eight major lineages based on marker expression. Panels show representative feature plots for EPCAM, PECAM1, CD3D, MS4A1, LYZ, and COL1A1. The distribution of mismatch repair–proficient (MMRp) and –deficient (MMRd) tumors across major cell types is also shown. [file peerj-14-20663-s002.png]

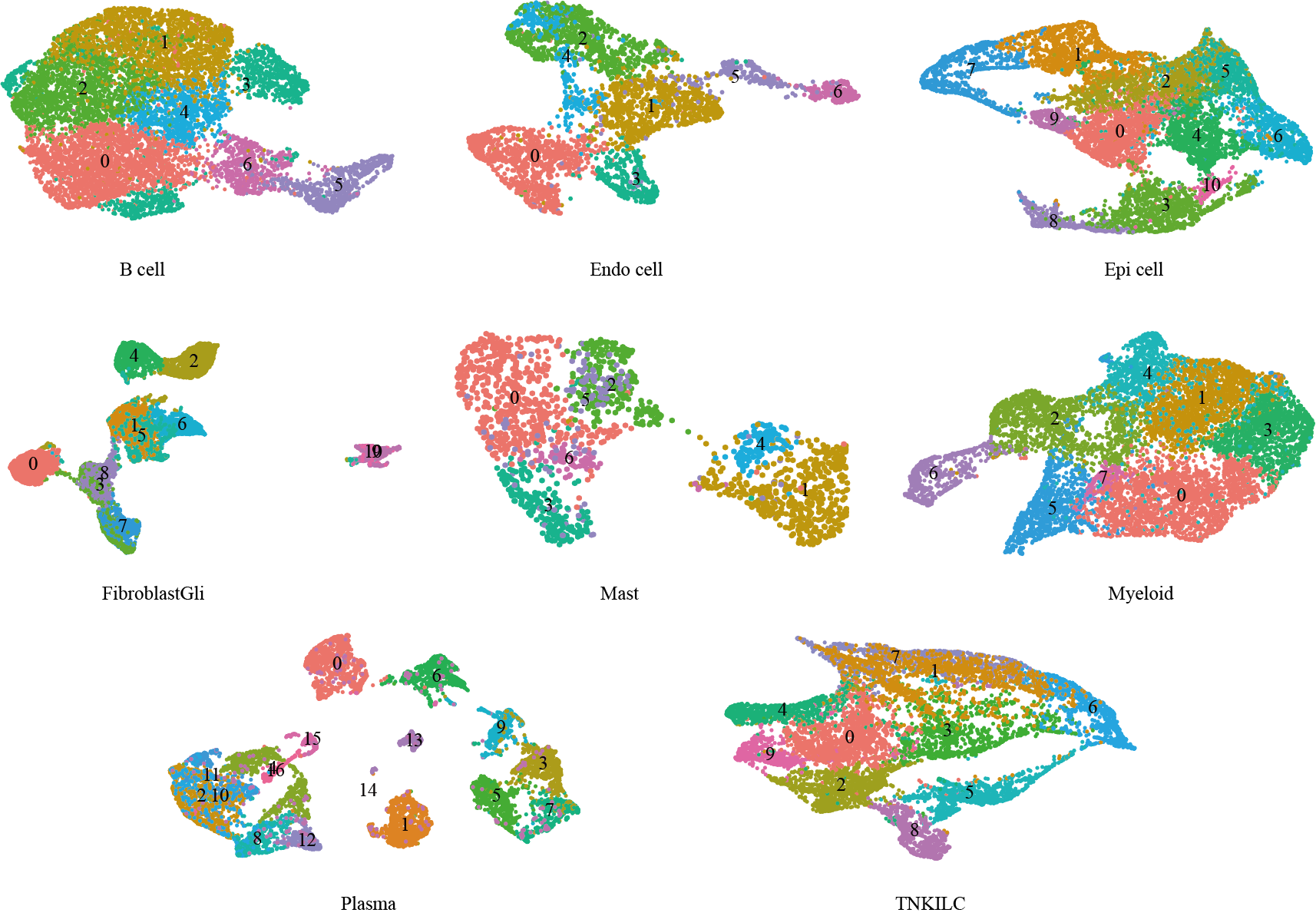

Supplement: Supplemental Information 3 — Non-negative matrix factorization (NMF)–based UMAP clustering within each major lineage identifies transcriptionally distinct subpopulations (e.g., Epi_1–Epi_10, Endo_1–Endo_6, Myeloid_1–Myeloid_5). Representative marker genes for each subpopulation are shown in heatmaps. [file peerj-14-20663-s003.png]

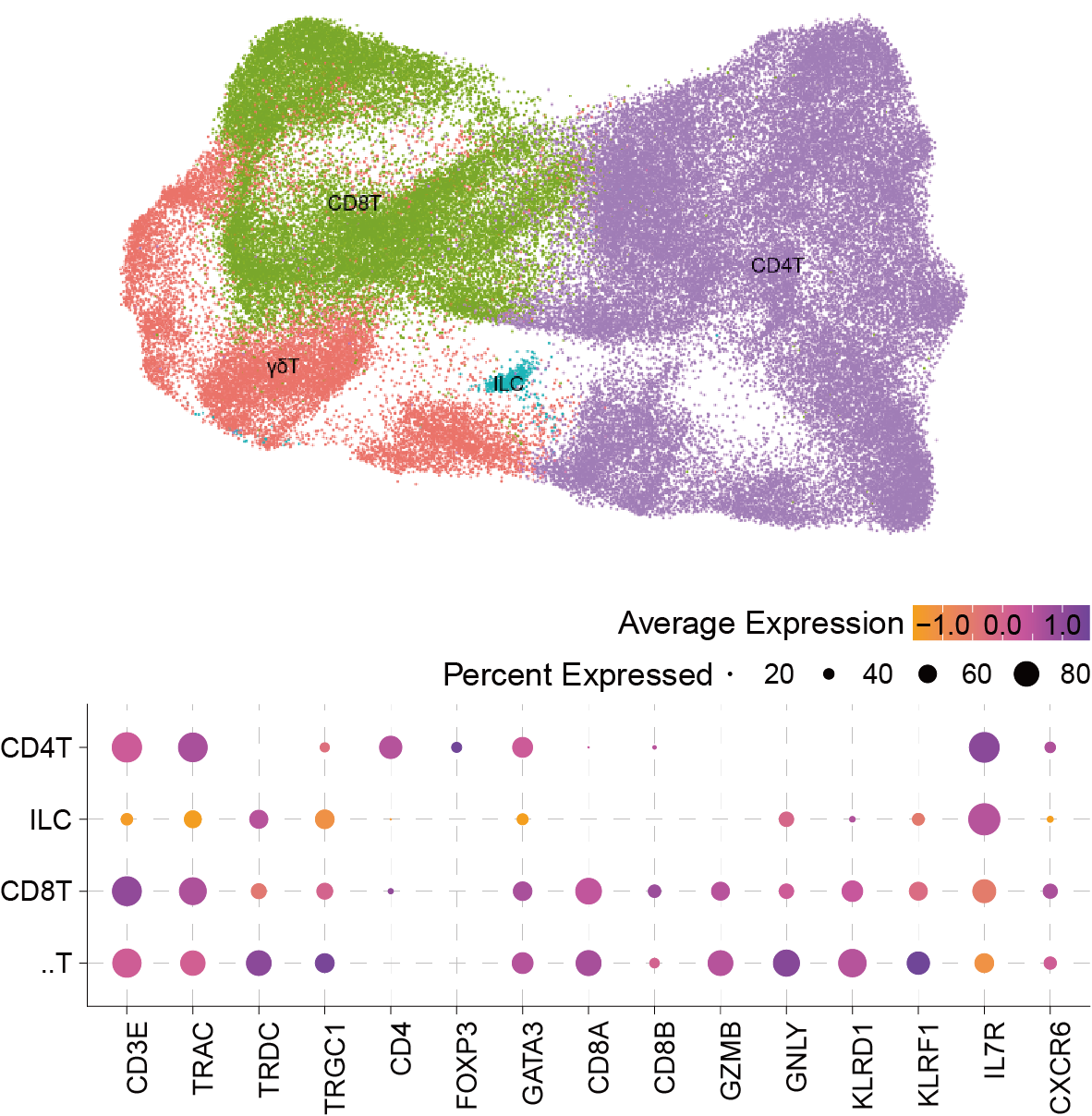

Supplement: Supplemental Information 4 — UMAP visualization and feature plots showing expression of CD4, CD8A, TRDC, NCR1, and IL7R, delineating CD4+ T cells, CD8+ T cells, γδ T cells, NK cells, and innate lymphoid cells (ILCs). CXCR6 expression is primarily enriched in the CD8+ T-cell subset. [file peerj-14-20663-s004.png]

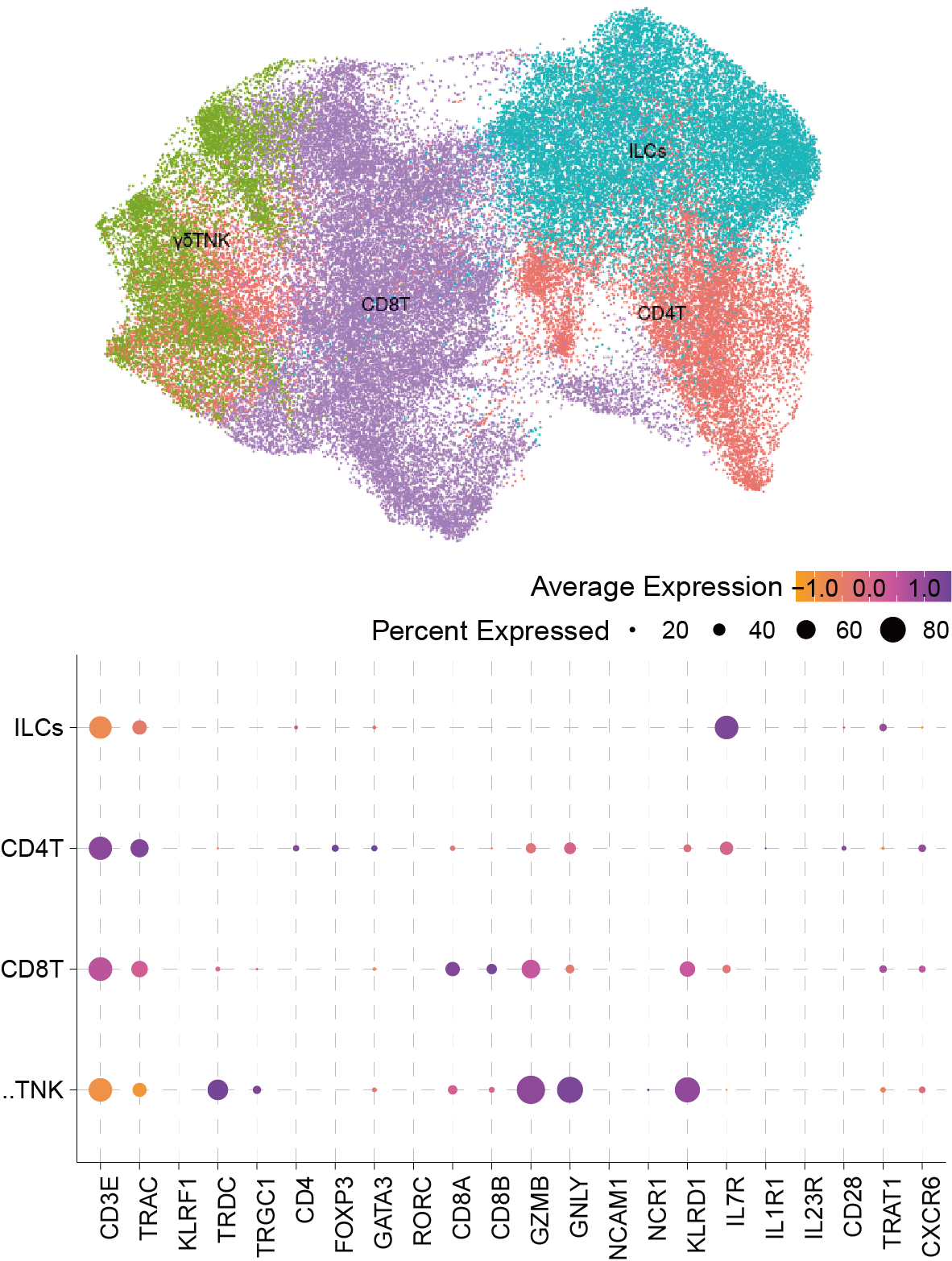

Supplement: Supplemental Information 5 — Violin and feature plots showing CXCR6 expression across tumor and normal tissues in MMRp and MMRd subgroups from GSE178341. The proportion of CXCR6+CD8+ T cells is elevated in tumors compared to adjacent normal tissue and higher in MMRd relative to MMRp tumors. [file peerj-14-20663-s005.png]

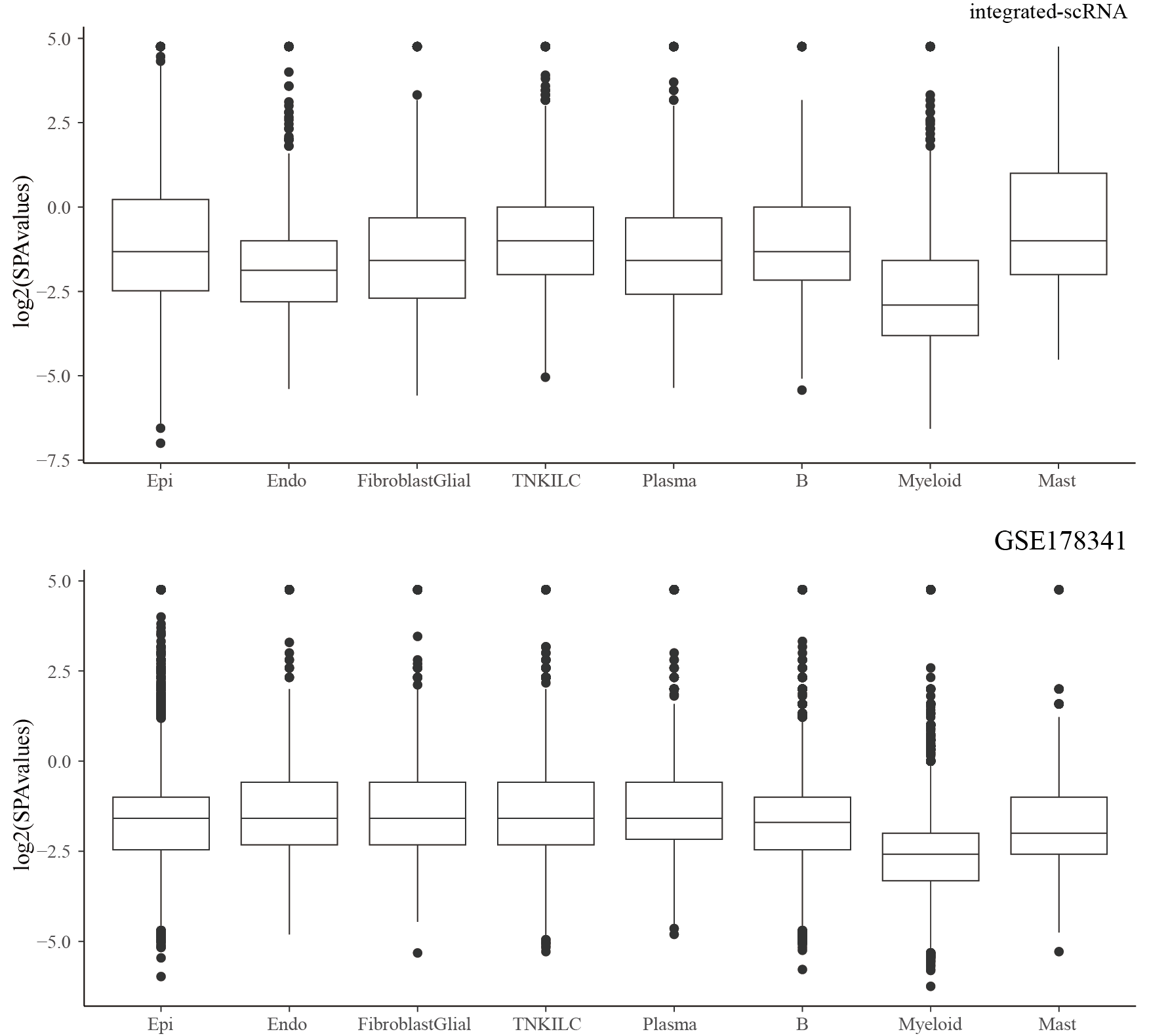

Supplement: Supplemental Information 6 — Boxplots showing SPA (intracellular accumulation) and Pi (pericellular accumulation) scores for putrescine across eight major cell types in the integrated scRNA-seq dataset. T/NK/ILC cells, epithelial cells, and mast cells exhibit the highest SPA values, while myeloid cells show the lowest. [file peerj-14-20663-s006.png]

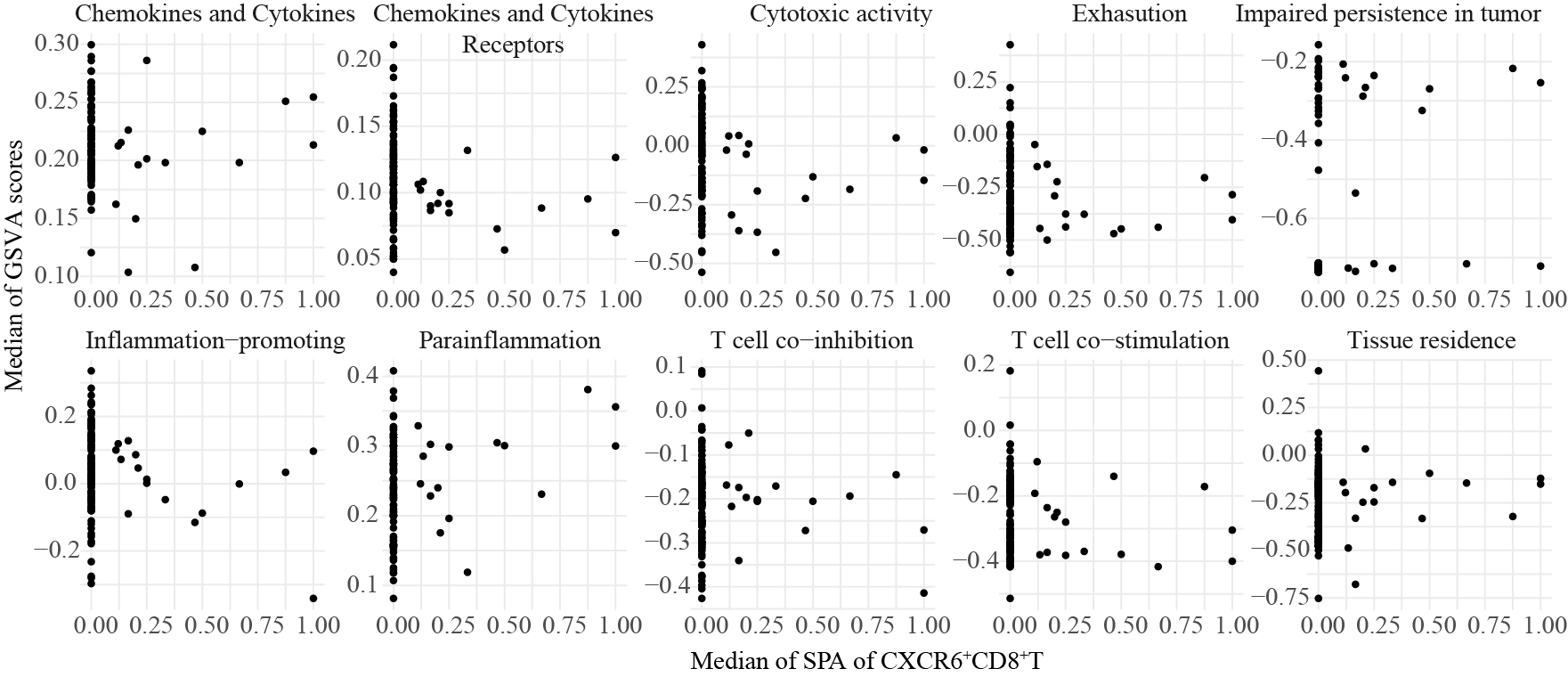

Supplement: Supplemental Information 7 — Spearman correlation analysis between Pi scores and GSVA enrichment scores of cytotoxicity, inflammation promotion, exhaustion, and co-inhibition pathways in CXCR6+CD8+ T cells. Higher pericellular putrescine levels negatively correlate with cytotoxic and inflammatory signatures, supporting functional suppression of CXCR6+CD8+ T cells. [file peerj-14-20663-s007.png]

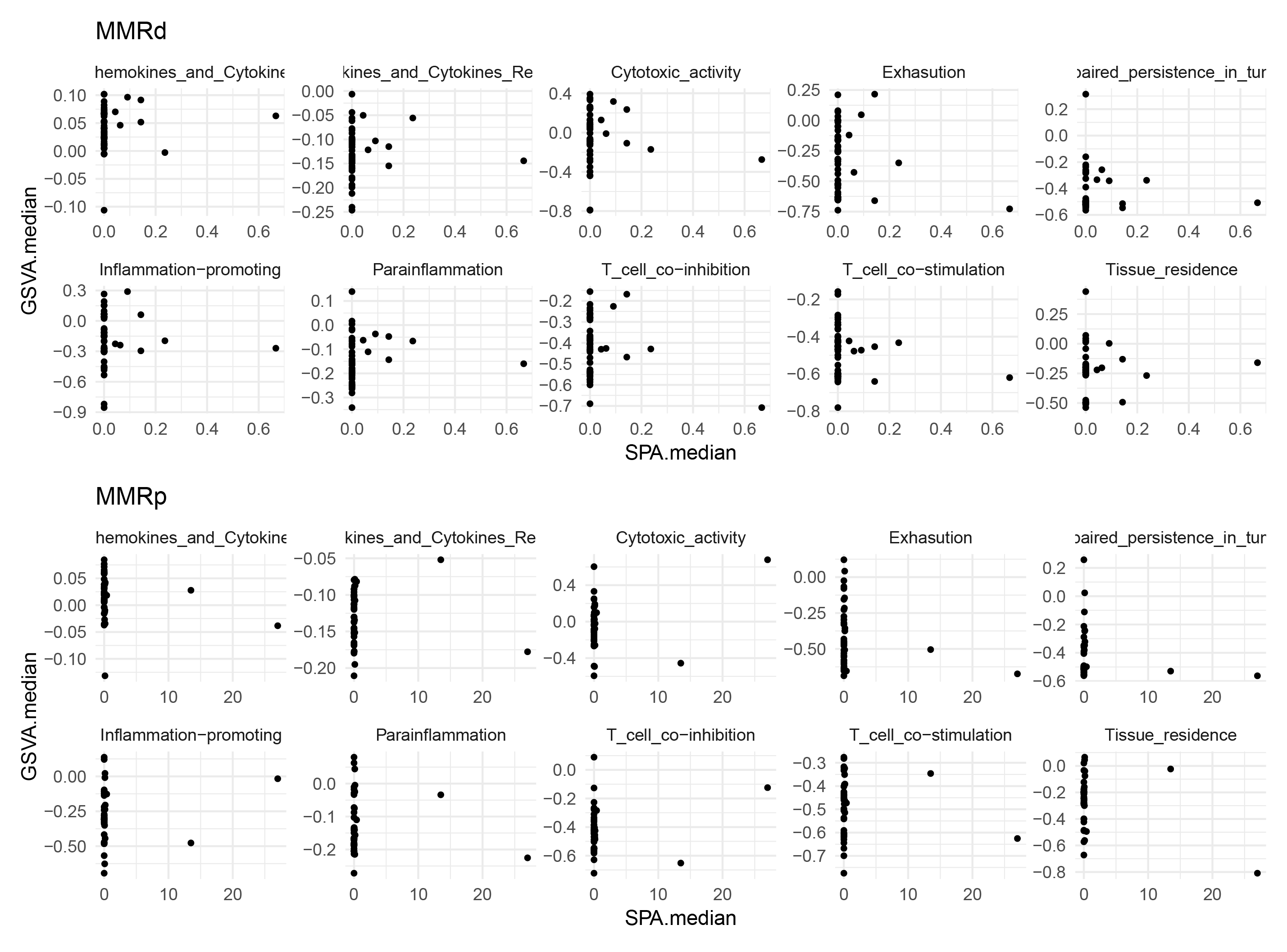

Supplement: Supplemental Information 8 [file peerj-14-20663-s008.png]

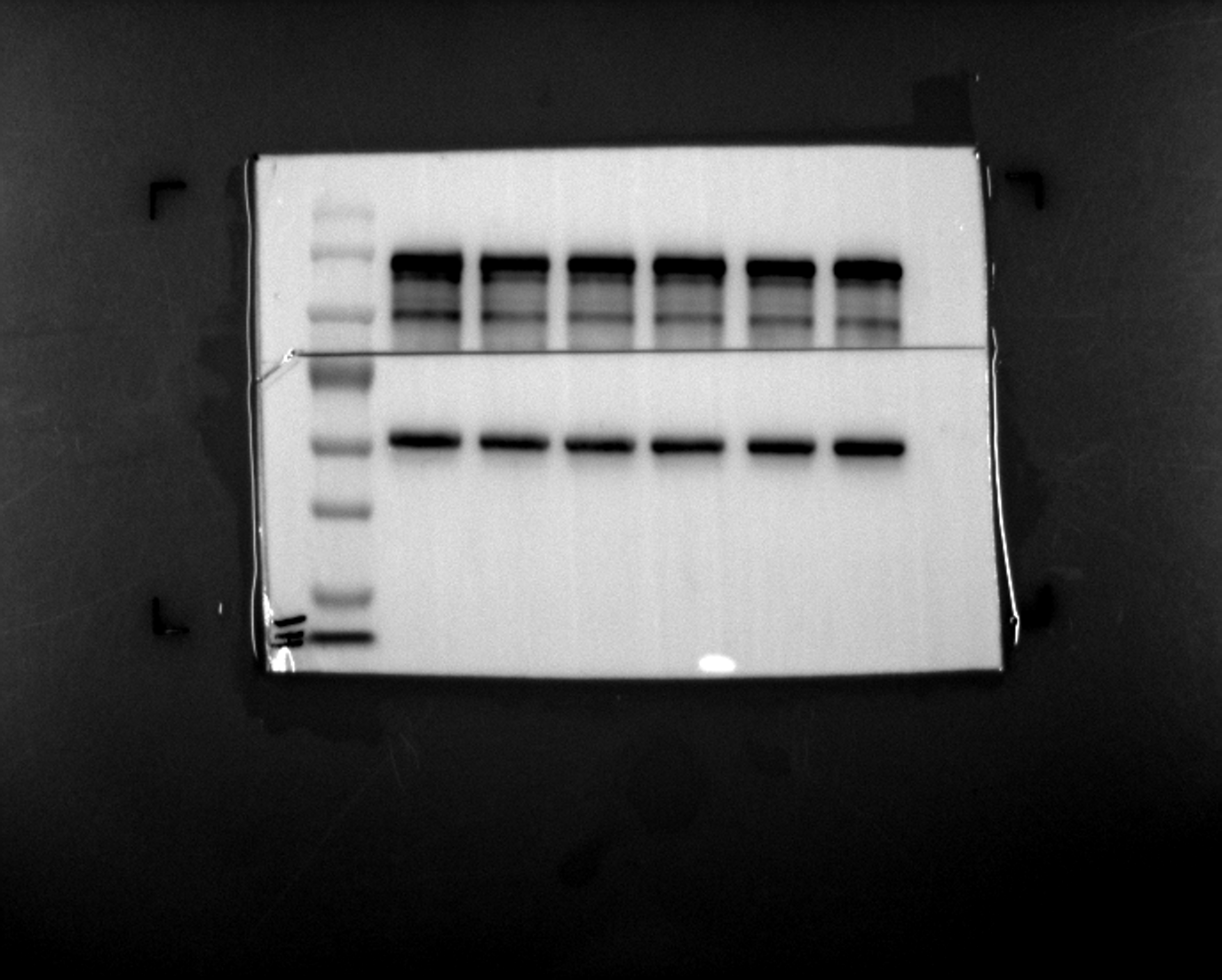

Supplement: Supplemental Information 13 [file peerj-14-20663-s013.zip › original WB/HCT116-E-cadherin.tif]

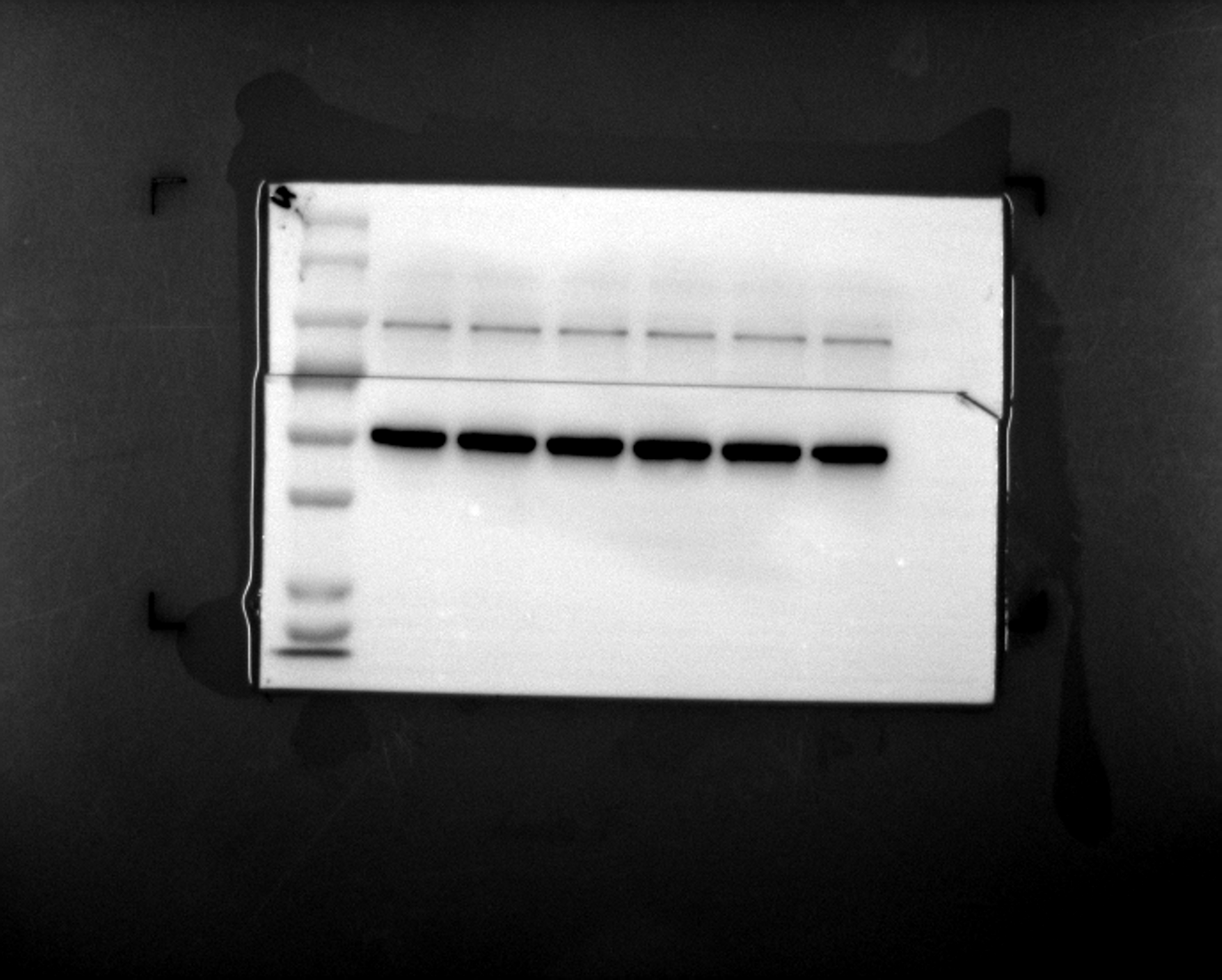

Supplement: Supplemental Information 13 [file peerj-14-20663-s013.zip › original WB/HCT116-N-cadherin.tif]

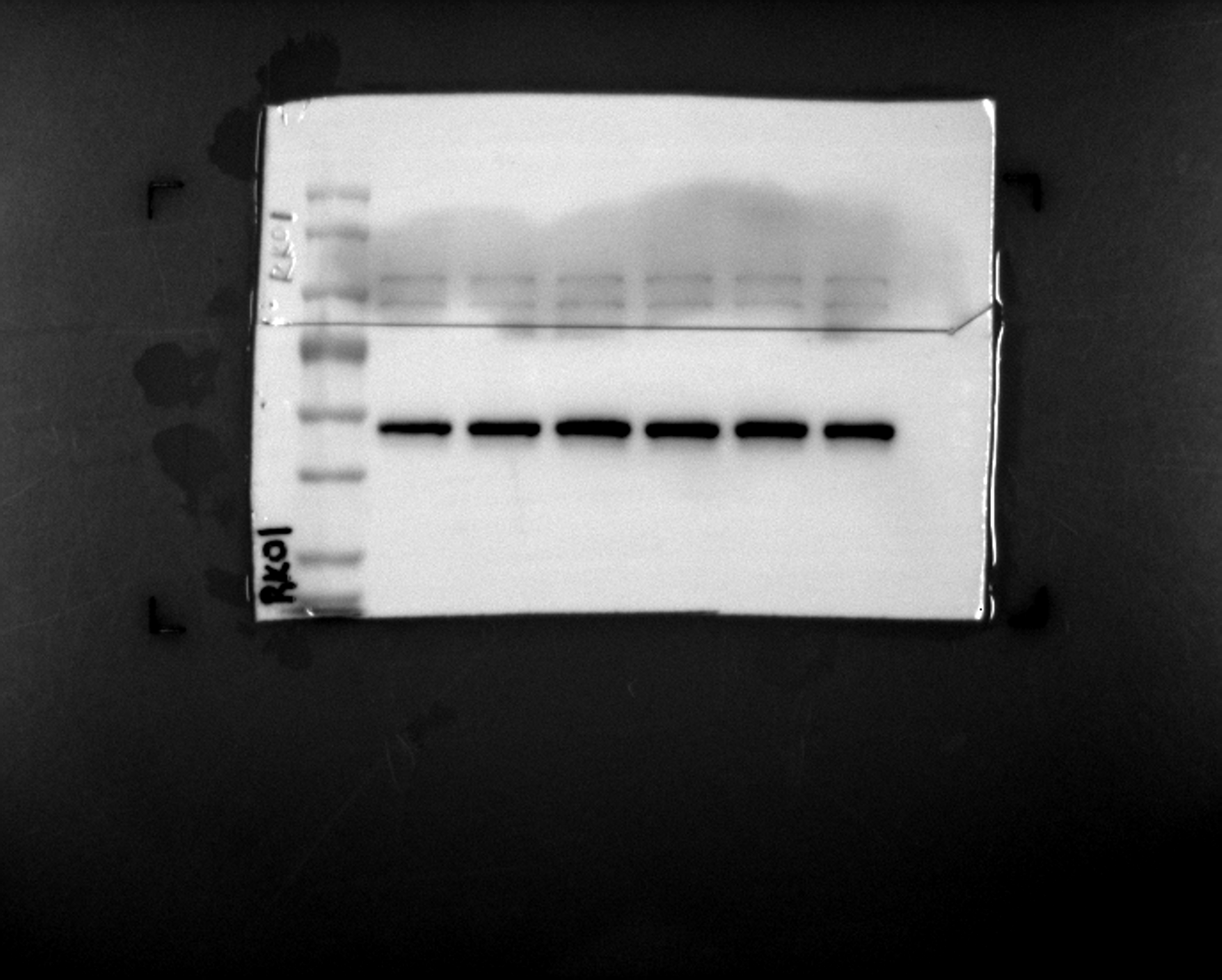

Supplement: Supplemental Information 13 [file peerj-14-20663-s013.zip › original WB/RKO-E-cadherin.tif]

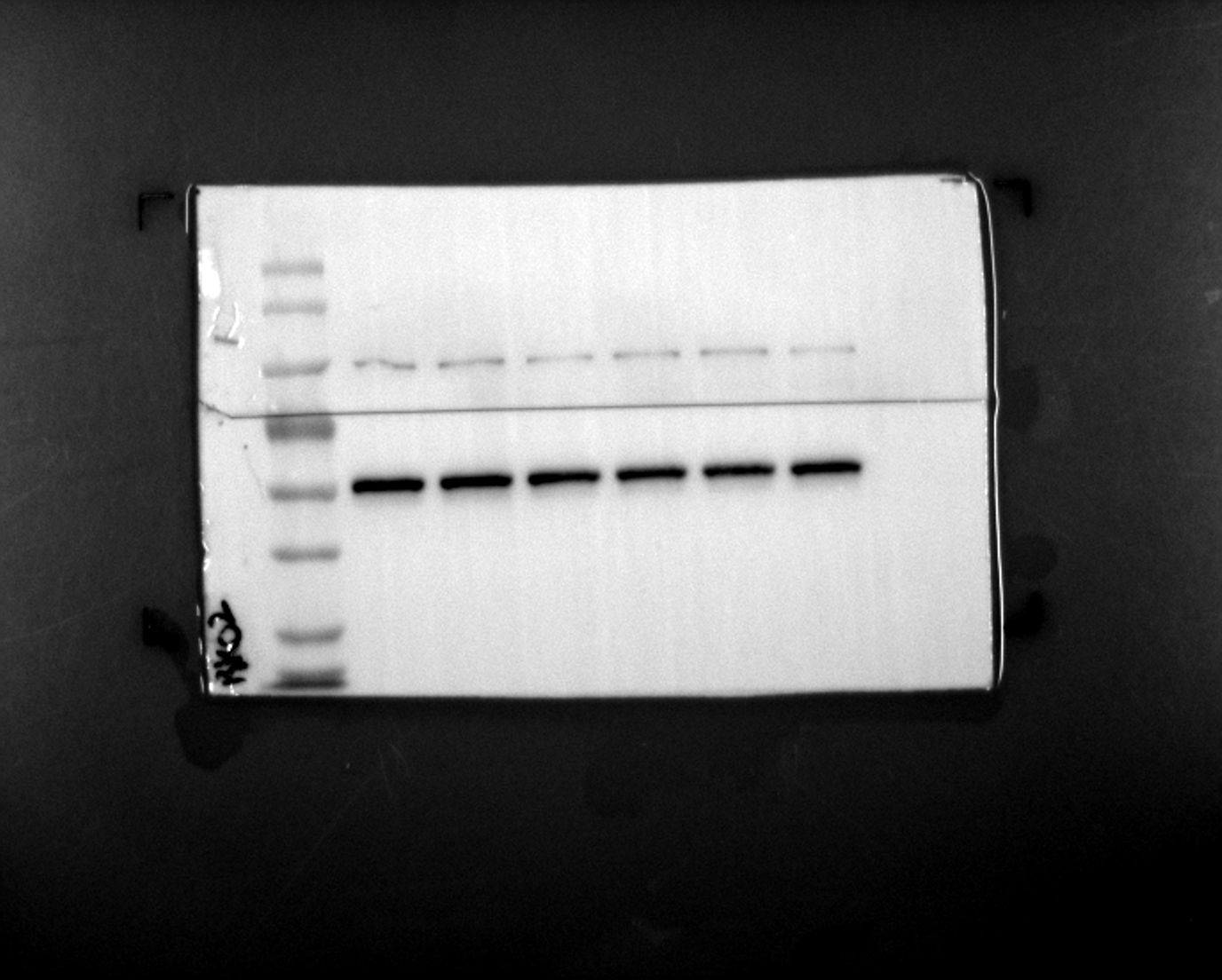

Supplement: Supplemental Information 13 [file peerj-14-20663-s013.zip › original WB/RKO-N-cadherin.tif]
